# Low-Volume Reaction Monitoring of Carbon Dot Light Absorbers in Optofluidic Microreactors

**DOI:** 10.1021/acscatal.3c02212

**Published:** 2023-06-26

**Authors:** Takashi Lawson, Alexander S. Gentleman, Ava Lage, Carla Casadevall, Jie Xiao, Tristan Petit, Michael H. Frosz, Erwin Reisner, Tijmen G. Euser

**Affiliations:** † NanoPhotonics Centre, Cavendish Laboratory, University of Cambridge, JJ Thomson Avenue, Cambridge CB3 0HE, U.K.; ‡ Yusuf Hamied Department of Chemistry, University of Cambridge, Lensfield Road, Cambridge CB2 1EW, U.K.; § Helmholtz-Zentrum Berlin für Materialien und Energy GmbH, Albert-Einstein-Straße 15, 12489 Berlin, Germany; ∥ Max Planck Institute for the Science of Light, Staudtstr. 2, 91058 Erlangen, Germany

**Keywords:** photocatalysis, microreactors, carbon dots, optofluidics, laser spectroscopy, hollow-core photonic crystal fibers

## Abstract

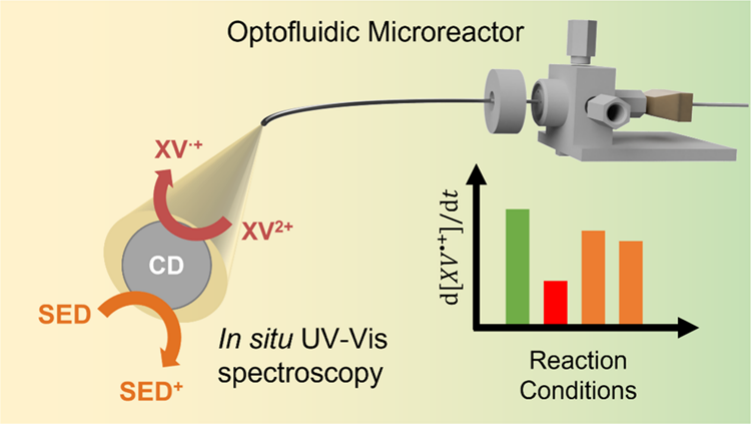

Optical monitoring and screening of photocatalytic batch reactions using cuvettes *ex situ* is time-consuming, requires substantial amounts of samples, and does not allow the analysis of species with low extinction coefficients. Hollow-core photonic crystal fibers (HC-PCFs) provide an innovative approach for *in situ* reaction detection using ultraviolet–visible absorption spectroscopy, with the potential for high-throughput automation using extremely low sample volumes with high sensitivity for monitoring of the analyte. HC-PCFs use interference effects to guide light at the center of a microfluidic channel and use this to enhance detection sensitivity. They open the possibility of comprehensively studying photocatalysts to extract structure–activity relationships, which is unfeasible with similar reaction volume, time, and sensitivity in cuvettes. Here, we demonstrate the use of HC-PCF microreactors for the screening of the electron transfer properties of carbon dots (CDs), a nanometer-sized material that is emerging as a homogeneous light absorber in photocatalysis. The CD-driven photoreduction reaction of viologens (XV^2+^) to the corresponding radical monocation XV^•+^ is monitored *in situ* as a model reaction, using a sample volume of 1 μL per measurement and with a detectability of <1 μM. A range of different reaction conditions have been systematically studied, including different types of CDs (*i.e.*, amorphous, graphitic, and graphitic nitrogen-doped CDs), surface chemistry, viologens, and electron donors. Furthermore, the excitation irradiance was varied to study its effect on the photoreduction rate. The findings are correlated with the electron transfer properties of CDs based on their electronic structure characterized by soft X-ray absorption spectroscopy. Optofluidic microreactors with real-time optical detection provide unique insight into the reaction dynamics of photocatalytic systems and could form the basis of future automated catalyst screening platforms, where samples are only available on small scales or at a high cost.

## Introduction

Carbon dots^1,2^ (CDs) are quasi-spherical particles, generally below 10 nm in size,^3,4^ which can exist in two forms: crystalline with sp^2^ character (graphitic and nitrogen-doped graphitic, gCDs and NgCDs, respectively) or amorphous (aCDs) with a majority contribution of sp^3^ carbons. They are proposed as potential next-generation photosensitizers as they offer a unique combination of properties. They are robust, environmentally benign, visible-light-active, biocompatible, and stable.^5^ CDs are surface-functionalized with oxygen-containing functional groups such as carboxyls and alcohols, enabling exceptional water solubility and the ability to tune their surface functionalities.^2,6,7^ Moreover, CD synthesis is inexpensive, simple, and scalable, and can even be produced from waste such as lignocellulosic biomass.^2,8^

When implemented in photochemical processes, photoexcited CDs can undergo electron transfer in the presence of an electron acceptor (EA), such as viologens or molecular catalysts,^9,10^ or an electron donor. Viologen redox couples are established electron relays in a range of hydrogen-evolving systems.^11,12^ Strauss et al. have postulated that CDs and viologens form preorganized bound complexes in solution due to the electrostatic attraction between the negatively charged CD surface and viologen dication, which results in a decrease in the measured ζ-potential.^13^ Sacrificial electron donors (SEDs) are often used in place of an oxygen evolution counter reaction to simplify the study of a photocatalytic half-reaction. As such, the ideal SED should neither rate-limit the desired reaction nor interfere with the reductive chemistry. Commonly used SEDs in solar fuel synthesis include tertiary amines such as ethylenediaminetetraacetic acid (EDTA), triethylamine (TEA), and triethanolamine (TEOA), which form known noninnocent species upon oxidation^14^

The combination of CDs with synthetic Ni- and Co-based molecular catalysts in hybrid photocatalytic systems has been shown to evolve hydrogen in water,^8,9,15^ and the use of CDs has been reported in organocatalysis, for example, acid–base catalysis, hydrogen-bond catalysis, and aminocatalysis.^16−19^ Graphitization of CDs enhances light absorption, and nitrogen doping of graphitic CDs (NgCDs) can prolong the lifetime of photogenerated charges, thereby enhancing their overall photocatalytic performance.^5^

Despite the advantages of CDs, the quantum yield of CD-based systems is relatively low and the photophysical properties are not fully understood. Understanding and optimization of these photocatalytic systems require comprehensive knowledge of the reaction kinetics that can only be obtained through screening of different types of CDs, EAs, SEDs, and other reaction conditions. Unfortunately, screening processes for photocatalysts are challenging as they typically rely on *ex situ* methods with high sample concentrations for analysis, which may not necessarily reflect the system under real catalytic conditions and therefore lead to poor reaction optimization decisions. Additionally, the paucity of detailed cuvette-based studies aimed at varying reaction parameters for photocatalytic reaction optimization highlights the cumbersome nature of performing such investigations, with relatively large (mL) sample volumes, long sampling intervals, and difficulty in modulating the irradiance. As such, there is a growing need for microreactors that allow rapid changes in reaction conditions to be monitored *in situ* within tiny reaction volumes and with enhanced time resolution.^20,21^

Hollow-core photonic crystal fibers (HC-PCFs) have emerged as an attractive optofluidic platform for *in situ* monitoring within microreactors. A key advantage is that they allow light to be guided along a microfluidic channel over extended lengths while keeping the internal sample volume very low. HC-PCFs thus enable highly sensitive liquid-phase spectroscopy on sub-μL sample volumes.^22−29^ The dead volume, accounting for tubing and the pressure cell in this system, is <55 μL. Once a measurement is completed, a fresh sample can infiltrate the fiber by flowing *ca.* 10 μL of the sample through the fiber (corresponding to 10× the internal volume contained in the fiber core). The dead volume can be minimized to below 1 μL by replacing the pressure cell with a microfluidic chip.^30^

We have previously demonstrated that HC-PCF microreactors can be used to monitor *in situ* CD-driven photoreduction of viologens (XV^2+^) to the radical monocation XV^•+^ on volumes less than 36 nL.^31^ Alternative types of hollow waveguides, such as a large-diameter (>800 μm) Teflon-coated capillary fiber,^32−34^ have also been used for reaction monitoring. However, homogeneous excitation of strongly absorbing CD samples can only be achieved by side-excitation, requiring the waveguide to be made of ultraviolet (UV)-transparent materials such as fused silica.

**Figure 1:**
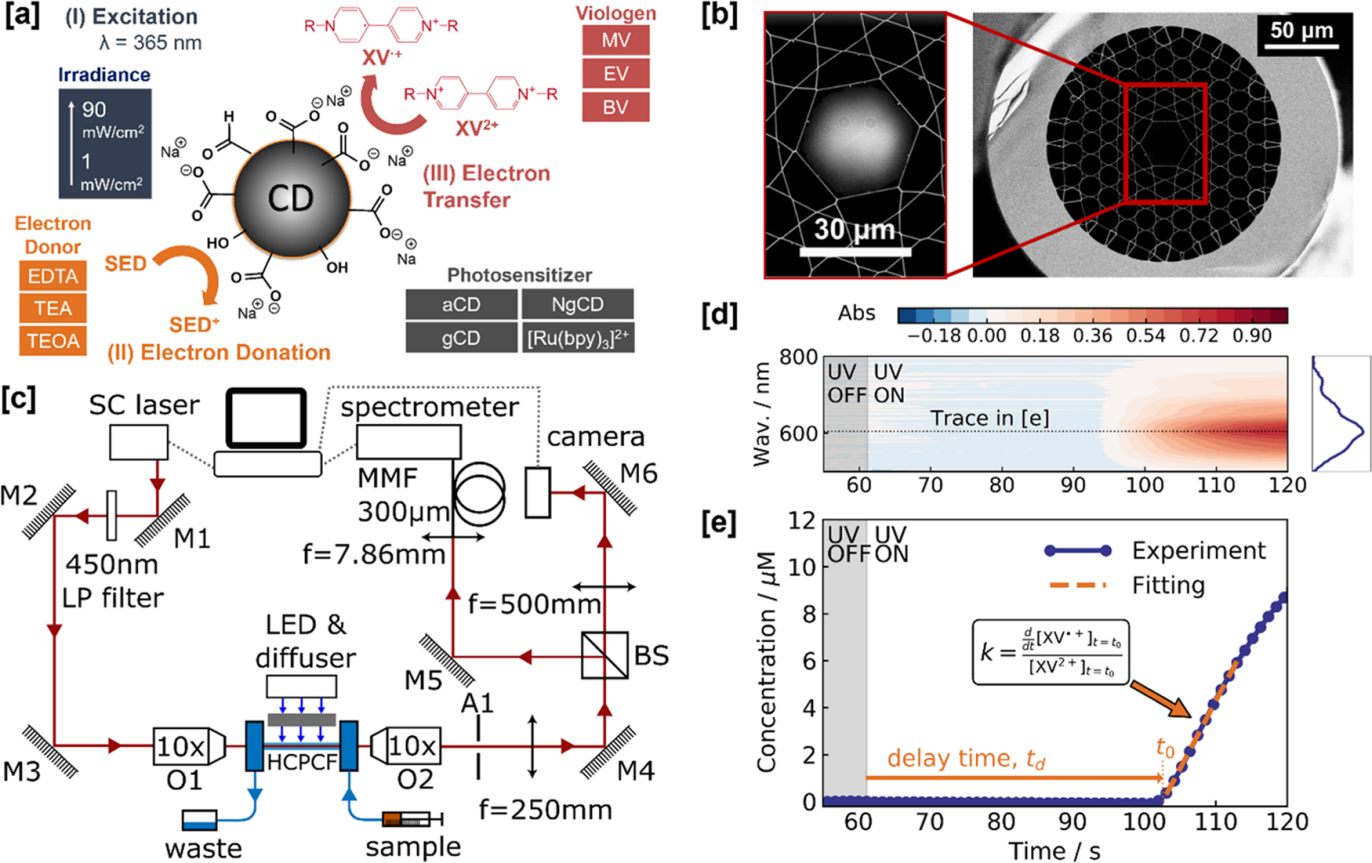
Fiber-based CD screening. [a] Schematic of CD-driven photoreduction of viologens and the four screening factors explored in this study: the irradiance, sacrificial electron donor (SED) used, CD photosensitizer type, and viologen species (XV). [b] Scanning electron microscopy (SEM) image of the core region of the kagomé-style hollow-core photonic crystal fiber (HC-PCF) overlaid with the measured mode intensity profile (450–600 nm). [c] Optical setup enabling the transmitted mode to be simultaneously analyzed by a camera (see [b]) and its spectrum by a spectrometer (see [d]). BS, beamsplitter; LP, long-pass; MMF, multimode fiber (300 μm core diameter); SC, supercontinuum. O1 corresponds to the in-coupling objective and O2 corresponds to the out-coupling objective. A1 is an aperture used for alignment. [d] Time-resolved spectra are converted to absorbance with a trace taken at 600 nm to deduce the XV^•+^ concentration in [e]. The spectral shape of the absorbance profile is characteristic of an XV^•+^ species. [e] Fitting method for calculating the delay time, *t_d_*, and photoreduction rate constant, *k*. The time at which the electron transfer starts, *t*_0_, is defined by the *x*-intercept of the linear fitting function.

Here, we build upon our previous proof-of-principle study and use UV–visible (UV–vis) spectroscopy performed with HC-PCF microreactors to demonstrate the ability to screen for a wide range of reaction conditions (Figure 1a), generating new kinetic insights into the photoactivity of CDs and improving our understanding of structure–function relations. We use viologens as indicators of electron transfer from CDs due to their distinctive UV–vis absorption peaks and their ability to provide insight into single electron transfers. CD-Viologen reactions thus form an excellent test system to understand redox mechanisms independently from catalysis requirements. The performance of CD light absorbers is benchmarked against the conventional photosensitizer [Ru(bpy)_3_]^2+^ (bpy = 2,2′-bipyridine), with the electron transfer properties of each CD type also discussed, as characterized by soft X-ray absorption spectroscopy (XAS). This work demonstrates how fiber-based optofluidic microreactors can provide comprehensive kinetic insight into an exemplar photocatalytic platform, *i.e*. CDs, which hold promise for driving a wide range of sustainable organic transformations.^16,35^

## Carbon Dot Screening Scheme

The proposed anaerobic reaction mechanism is summarized in eqs 1–6 and is based on studies by Mandal et al. and Suzuki et al. who used [Ru(bpy)_3_]^2+^ as a photosensitiser.^36,37^
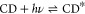
1
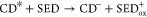
2
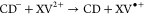
3
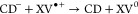
4
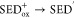
5
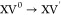
6

Reaction 1 represents the formation of the photoexcited carbon dot, CD*, which is then reductively quenched by a SED in reaction 2 according to the literature.^14,15^ This results in a reduced carbon dot, CD^–^, and an oxidized SED species, SED_ox_^+^, whereby the SED_ox_^+^ ideally comprises innocent decomposition products after electron transfer. Reaction 3 represents the electron transfer from CD^–^ to XV^2+^ to form a radical monocation that we can detect by UV–vis absorption spectroscopy (λ_max_ = 600 nm). Reaction 4 represents an electron transfer from CD^–^ to XV^•+^ to form a neutral XV^0^.^37^ The SED_ox_^+^ species, in the cases of EDTA, TEA, and TEOA, refers to the aminyl radical species, R_2_N^•+^CH_2_–, which is readily deprotonated to form SED′, a carbon-centered radical species and strong reducing agent, R_2_NC^•^H–,^36^ in reaction 5. XV^0^ is readily protonated and reacts with water to form XV′, a decomposition product without a UV–vis absorbance feature in reaction 6.^38^

There are a few pathways that can lead to the depletion of XV^•+^. For instance, the concentration of XV^•+^ can be depleted in interaction with CD^–^ through a second electron transfer in reaction 4 or with highly reducing decomposed SED (SED′). Another possible route for XV^•+^ depletion is *via* direct interaction with non-photoexcited and nonreduced CDs; a pathway not considered in the proposed reaction mechanism. Evidence for this pathway is presented in the Results and Discussion section.

## Experimental Section

### Hollow-Core Photonic Crystal Fiber (HC-PCF)

This study utilizes kagomé-style HC-PCFs (Figure 1b), whose guidance properties are well described by the anti-resonant reflection theory.^39,40^ By controlling the thickness of the glass struts surrounding the hollow-core cladding structure, this guidance mechanism permits broadband transmission extending from the ultraviolet (UV) to near-infrared, and hence, kagomé-style HC-PCFs are the preferred choice for UV–vis spectral measurements.^41^ The fiber used in this study was fabricated with a strut thickness, *t*, of 160 nm to suppress the first resonant loss, λ_1_, to 183 nm (see eq 7).^40^ The first resonant loss was calculated for a fiber infiltrated with water, assuming a glass refractive index, *n*_glass_, of 1.45. Additionally, a core region of 30 μm diameter facilitated a UV–vis detection volume of *ca.* 7 nL cm^–1^.
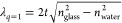
7

Kagomé-style HC-PCFs were interfaced with standard polyether ether ketone (PEEK) microfluidic tubing through custom-designed stainless steel pressure cells with sapphire windows (see Supporting Information, Figure S1), enabling the HC-PCF to be loaded with a liquid solution while remaining optically accessible. HC-PCFs were sealed with PEEK sleeves (IDEX F-240 Blue) and finger-tight fittings, as in previous work.^31^ Samples were thoroughly purged under nitrogen for at least 15 min before loading into the fiber and inserted through the out-coupling facet to minimize disturbances to the optical coupling. All samples were injected into the pressure cell using gas-tight syringes and a syringe pump (Aladdin AL-1000), having passed through 0.2 μm pore size cellulose acetate filters to prevent fiber blockages.

Repeat measurement runs were loaded successively into the fiber and started once the spectrum returned to the reference baseline. This ensured that residual reaction products from the previous run had been pushed clear of the fiber. The fiber was cleaned with 200 μL of water (200× the internal fiber volume) between sample sets of different reaction conditions. Subsequent samples were loaded once the spectrum matched an internal water reference.

### UV–Vis Absorption Spectroscopy Setup

A broadband fiber-coupled supercontinuum (SC) laser (NKT Photonics SuperK Compact—Figure 1c) was used to perform UV–vis absorption spectroscopy. A 450 nm long-pass filter was placed in the probe beam path to prevent excitation of the photochemical reaction by the probe light. Furthermore, the probe power coupled into the fiber was reduced to 2.2 μW using neutral density filters. The beam was launched into the core of the liquid-filled fiber, exciting a fundamental optical mode (Figure 1b). We achieve transmission of 48%T at 600 nm for 20 cm of fiber infiltrated with water, indicating a good coupling efficiency (see Supporting Information, Figures S2 and S3).

A beamsplitter (BS) cube divides the transmitted probe light over an imaging CCD camera (IDS UI-3240LE-NIR-GL) and a fiber-coupled spectrometer (Ocean Optics QE Pro 65000). The guided light was strongly confined to the core. An aperture (A1) was placed in the optical setup to aid optical alignment.

UV–vis absorption spectra were generated by dividing the spectrometer counts by a reference spectrum, which in this case is the spectrum of the initial unirradiated sample (Figure 1d). Concentration was then calculated from the absorption peak at 600 nm *via* the Beer–Lambert law (Figure 1e), as detailed in the Supporting Information. Experiments reveal a delay time, *t*_*d*_, between the excitation source being switched on and the formation of XV^•+^. We have previously reported on this finding.^31^

A 365 nm UV light-emitting diode (LED) source (HepatoChem EvoluChem 365PF) and an optical diffuser (Thorlabs DG20-220-MD) were used to side-irradiate a 5 cm section of the HC-PCF, ensuring a homogeneous excitation profile across the 36 nL UV–vis detection volume. UV excitation was chosen due to the significant absorption of CDs at 365 nm (see Supporting Information, Figure S4). Significantly higher concentrations of CDs would be required for irradiation at visible wavelengths, which would, in turn, reduce transmission of the broadband probe light, resulting in much noisier UV–vis data. The irradiance was varied between 1.6 and 88.4 mW cm^–2^ by placing 2″ × 2″ absorptive neutral density filters (Thorlabs NE2 series) between the excitation source and fiber. A 450 nm blue LED source (HepatoChem EvoluChem 450PF) was additionally utilized to benchmark the performance of nitrogen-doped graphitic carbon dots (NgCDs) against [Ru(bpy)_3_]^2+^ at an irradiance of 99.1 mW cm^–2^.

### X-ray Absorption Spectroscopy (XAS)

Experiments were conducted at the U49-2_PGM1 soft X-ray beamline of the BESSY II synchrotron using the LiXEdrom endstation. The CDs were drop-cast on a conductive Si substrate, and XAS was acquired in vacuum using total electron yield (TEY) mode detecting the sample drain current by a Keithley 6514 ammeter at the C and O K-edge and total fluorescence yield (TFY) collecting emitted photons by a photodiode placed in front of samples at the N K-edge.

## Materials

All chemicals and reagents were purchased from commercial suppliers and used as received unless otherwise noted. Laboratory-grade reagents were used for synthesis, and chemicals for the analytical part were of the highest available purity. Phosphate buffer solutions (0.2 M) and SED stock solutions (0.2 M) were prepared and verified using a pH electrode (Mettler Toledo, FiveEasy Plus) at pH 6 and pH 8. The pH of SED stock solutions was adjusted using sodium hydroxide and hydrochloric acid. Methyl viologen (MV^2+^), ethyl viologen (EV^2+^), and benzyl viologen (BV^2+^) stock solutions were made up by dissolving methyl viologen dichloride, ethyl viologen dibromide, and benzyl viologen dichloride salts, respectively. A stock solution of [Ru(bpy)_3_]^2+^ was made up by dissolving tris(bipyridine)ruthenium(II) chloride. All stock solutions were made up using Milli-Q purified water.

### Synthesis of CDs

All CD types were synthesized and characterized according to previously reported procedures.^5,9^ In brief, amorphous carbon dots (aCD) were synthesized by pyrolysis of citric acid at 180 °C for 40 h and graphitic carbon dots (gCD) were similarly synthesized by pyrolysis of citric acid at 180 °C for 40 h followed by pyrolysis at 320 °C for a further 100 h. Nitrogen-doped graphitic carbon dots (NgCD) were obtained by pyrolysis of aspartic acid at 320 °C for 100 h. To enhance water solubility, unless otherwise noted, all CD types were neutralized to pH 7 using NaOH and then freeze-dried to obtain the final product as a brown powder. All CDs were characterized by UV–vis, Fourier transform infrared (FTIR), and Raman spectroscopy (see Supporting Information, Figures S4 and S5).

## Results and Discussion

To ascertain whether XV^•+^ interacts with the CDs, MV^2+^ (160 μM) was chemically reduced to MV^•+^ with one equivalent of sodium dithionite (SDT, Na_2_S_2_O_4_) under nitrogen in a gas-tight cuvette. Further reduction of MV^•+^ to MV^0^ by Na_2_S_2_O_4_ is excluded based on reduction potentials (*E*^MV^•+^/MV^0^^ = −1.15 V *vs* normalized hydrogen electrode (NHE) and *E*^SDT/SDT^+^^ = −0.66 V *vs* NHE). Solutions were then prepared with and without the presence of a SED (*i.e*., EDTA). Subsequent titration with aCDs comprising different surface moieties (NMe_2_^+^, COO^–^, and COOH) under nitrogen revealed a reduction in the intensity of the characteristic UV–vis absorption band of MV^•+^ upon addition of small equivalents of the aCDs under nitrogen (see Supporting Information, Figure S7), with corresponding NMR data showing electron transfer occurring from MV^•+^ to the aCDs to yield MV^2+^ (see Supporting Information, Figure S8). This indicates that there may be an interaction with CDs that depletes the MV^•+^ concentration in solution when a SED is not present. The addition of the NMe_2_^+^-functionalized aCDs shows the slowest depletion rate of MV^•+^ compared to the COOH- and COO^–^-functionalized dots, which is most likely due to the electrostatic repulsive interactions between MV^•+^ and the positive surface charge on the NMe_2_^+^-functionalized aCDs.^42,43^

In comparison with titrations with the SED (EDTA) present, it is found that the SED forms a complex with MV^•+^, resulting in a smaller absorbance at 600 nm. Subsequent titration after the SED is added leads to a reversal in the trend of depletion rate, that is, positively charged NMe_2_^+^-functionalized aCDs deplete MV^•+^ faster than the negatively charged COO^–^-functionalized aCDs. This is most likely due to the cooperative electrostatic interactions of the negatively charged EDTA, which is expected for solutions of pH 4–7 (refer to Supporting Information, Table S1). The cooperative electrostatic interactions bring together MV^•+^ and the positively charged NMe_2_^+^-functionalized aCDs in solution, thus making them interact more quickly than the negatively charged COO^–^-functionalized aCDs. All forms of aCDs considered here are involved in the depletion of XV^•+^ and as such, an additional reaction for the proposed reaction mechanism must be considered reaction 8. It is likely that this reaction is mediated by the filling of trap states within the CDs, resulting in a sufficient driving force for this reaction to proceed.
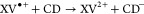
8

Following confirmation of this additional pathway to deplete XV^•+^, mechanistic insight into the photoactivity of CDs was obtained by the systematic screening of four variables (irradiance, photosensitizer, EAs, and SEDs), as shown in Figure 1a. A 20 cm length of kagomé-style HC-PCF was infiltrated with various aqueous solutions of CDs, viologens, and sacrificial electron donors (SEDs).

### Effect of aCD Concentration

**Figure 2:**
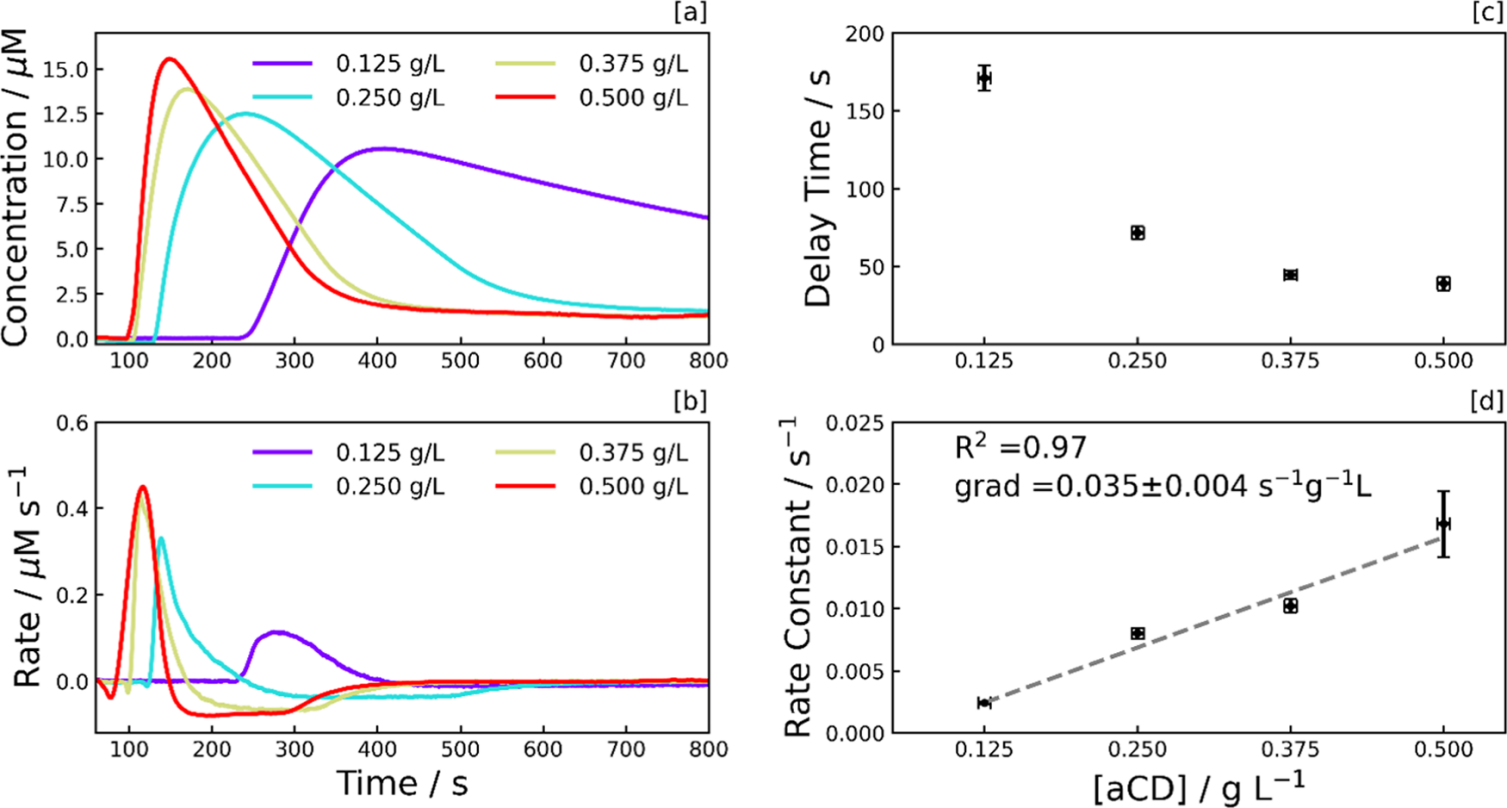
Photoreduction dependence on CD concentration. [a] MV^•+^ concentration, and [b] rate profiles, both illustrating the dependence of the reaction kinetics on amorphous carbon dot (aCD) concentration. The MV^•+^ concentration was calculated from the UV–vis absorption peak at 600 nm. Each sample consists of aCDs with 40 μM MV^2+^ and 0.1 M EDTA in an aqueous pH 6 phosphate buffer. Samples were initially kept in the dark and, from *t* = 60 s, were subject to continuous UV irradiation (λ = 365 nm) at an irradiance of 90.2 mW cm^–2^. [c] Delay time and [d] photoreduction rate constant, both plotted as a function of aCD concentration, with error bars signifying the standard deviation of triplicates.

Samples of 40 μM MV^2+^ and 0.1 M EDTA were prepared in an aqueous pH 6 phosphate buffer solution and infiltrated into the kagomé-style HC-PCF, with various concentrations of COO^–^-functionalized aCDs. These samples were subject to UV irradiation (λ = 365 nm, 90.2 mW cm^–2^), with the corresponding concentration and rate profiles shown in Figure 2a,b. As observed, peak radical monocation generation (λ_max_ = 600 nm) is higher when the CD concentration is greater, suggesting a dynamic equilibrium exists between the photoinduced rate of formation of the radical monocation and the depletion rate of the radical monocation. At higher concentrations of CDs, the position of the equilibrium is shifted toward the radical monocation given the higher [CD*]. However, a rise in [CD] is also associated with a rise in [CD^–^], with CD^–^ interacting with MV^•+^ as in reaction 4. This results in a greater depletion rate following the peak, as observed in Figure 2b.

The peak in the reaction profile (Figure 2) results from the competition between the generation of MV^•+^reaction 3 and its depletion reaction 4. As MV^2+^ is depleted during the reaction, the rate of formation of MV^•+^ falls. In contrast, the rate of formation of MV^0^ increases. This manifests as a relatively sudden and significant decrease in the rate of MV^•+^ depletion when MV^•+^ reaches a critical amount. As observed in Figure 2, such rate-switching behavior is expected to arise earlier as [CD] increases, as MV^2+^ is consumed faster via reaction 3 inevitably resulting in a critical value of MV^•+^ being reached more rapidly. The origin of this rate-switching behavior is further verified by the [MV^•+^] peak being reached much later when the pH is lowered (see Supporting Information, Figure S10), which is most likely due to the rate of reaction 2 decreasing with increased solution acidity, which is consistent with protonation of the SED lowering its ability to reduce photosensitisers.^44,45^

The delay time (Figure 1e), which is the period between the excitation source being switched on and the formation of XV^•+^, is inversely proportional to CD concentration (Figure 2c). This implies that the mechanism behind the delay time is dependent on the number of photons absorbed. Potentially, this phenomenon is driven by interactions between CDs, such as the collision-induced filling of trap states within the CDs with excited electrons from the valence band before the CDs can transfer electrons to MV^2+^.^31^ Additionally, the photoreduction rate is linearly proportional to the CD concentration, with each addition of 0.1 g L^–1^ of aCDs contributing 3.5 × 10^–3^ s^–1^ to the photoreduction rate constant (Figure 2d).

### Effect of MV Concentration

**Figure 3:**
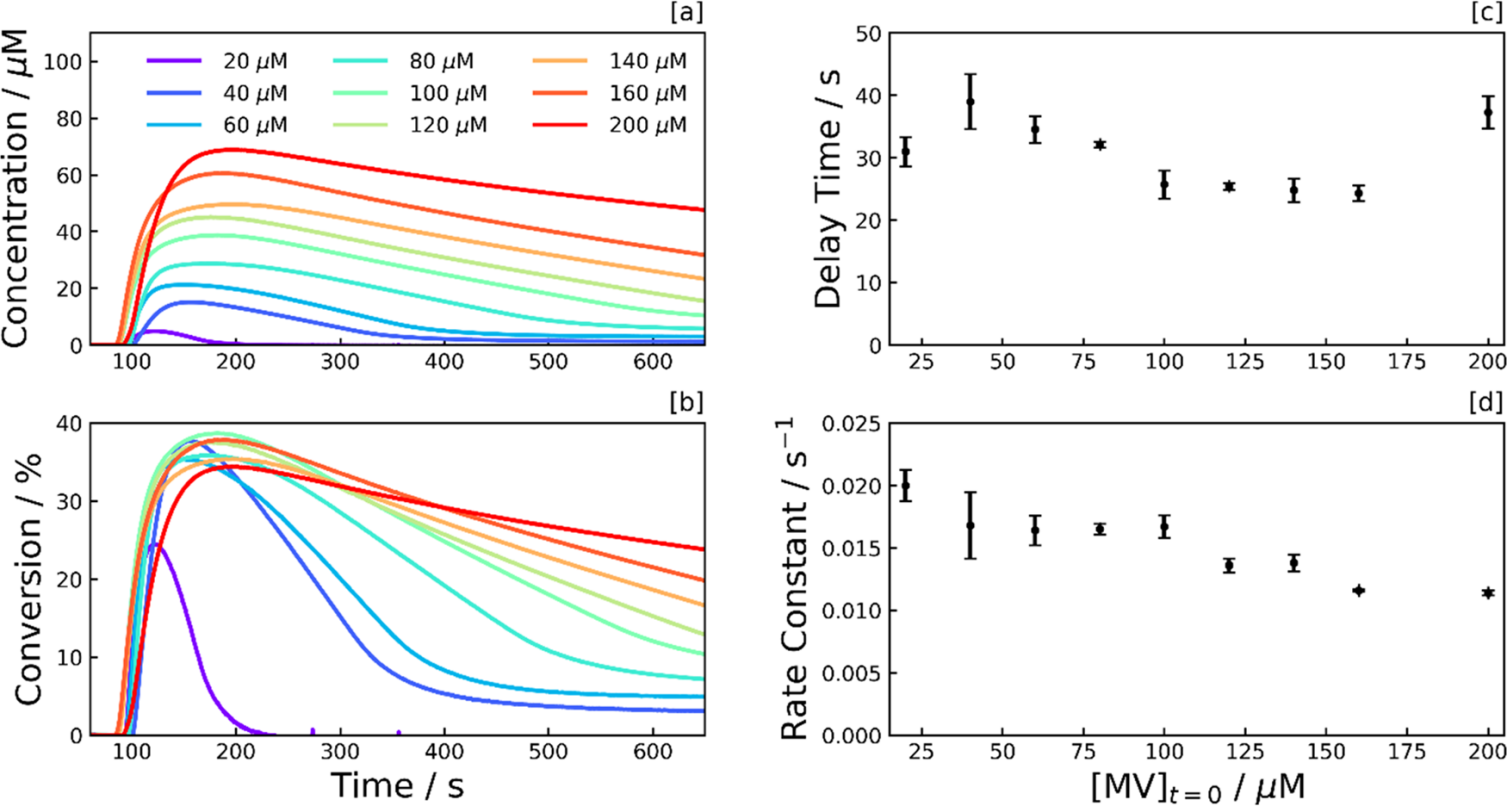
Photoreduction dependence on MV^2+^ concentration. [a] MV^•+^ concentration, and [b] percentage conversion profiles, illustrating the dependence of the reaction kinetics on the starting concentration of the methyl viologen dication, [MV^2+^]_*t*=0_. The MV^•+^ concentration was calculated from the UV–vis absorption tail at 670 nm. The conversion was calculated by dividing the concentration profile by the starting concentration of the methyl viologen dication. Each sample consists of MV^2+^ with 0.5 g L^–1^ aCDs and 0.1 M EDTA in an aqueous pH 6 phosphate buffer. Samples were subject to continuous UV irradiation (λ = 365 nm) from *t* = 60 s at an irradiance of 90.2 mW cm^–2^. [c] Delay time and [d] photoreduction rate constant, both plotted as a function of [MV^2+^]_*t*=0_, with error bars signifying the standard deviation of at least triplicates.

To provide further insight into the radical monocation depletion and delay time phenomenon, the starting concentration of MV^2+^, [MV^2+^]_*t*=0_, was varied to ascertain the point at which the aCD surface becomes saturated with pre-assembled MV species. This set of samples was made up of 0.5 g L^–1^ aCD and 0.1 M EDTA in an aqueous pH 6 phosphate buffer. Once samples were loaded into the fiber, they were subject to UV excitation as before, with the corresponding concentration and percentage conversion profiles shown in Figure 3a,b. Here, the greater concentration of MV^•+^ produced and corresponding strong UV–vis absorption meant that negligible transmitted counts were detectable at the spectrometer at 600 nm. Therefore, the conversion from absorbance to concentration was performed by averaging the absorbance within a 10 nm wide band around 670 nm, with a scaled absorption coefficient of 6.2 × 10^3^ M^–1^ cm^–1^ (see Supporting Information, Figure S11).

The depletion rate of MV^•+^ is constant at all starting concentrations of MV^2+^ (Figure 3a), suggesting that the CD^–^ species is rate-limiting the depletion. The rate equation takes the form of eq 9. If so, it suggests the rate of electron donation to the CDs from the SED is not coupled to the rate of electron transfer from the CDs to the MV^2+^, and we have similar amounts of CD^–^ at all starting concentrations of MV^2+^.
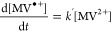
9

In contrast to the sweep in CD concentration, no clear correlation between [MV^2+^]_*t*=0_ and delay time is observed, signifying that the delay time phenomenon is not regulated by MV^2+^, but is intrinsic to the CDs themselves or dependent on the SED (Figure 3c). A drop in the photoreduction rate constant, *k*′, was observed above a starting concentration of 100 μM MV^2+^ (Figure 3d), with a maximum conversion to the radical monocation of 38% (Figure 3b). This drop indicates the switch to the photon-limited regime, where the local volumetric rate of photon absorption (*L*_p_, LVRPA) limits the photoreduction process. Above 100 μM MV^2+^, the photoreduction rate is no longer pseudo-first-order with respect to MV^2+^, and the LVRPA should be considered as part of the rate equation. This new second-order rate equation takes the form of eq 10.
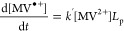
10

In general, a decrease in the photoreduction rate constant could be linked to the available surface area on each CD that MV^2+^ molecules can adsorb to. To give insight into this, we convert the mass concentration of aCDs to an approximate molar concentration, using their average diameter deduced by TEM (8 nm),^5^ together with their density, 1.5 g cm^–3^,^46^ yielding a molar concentration of 2 μM. This indicates that each carbon dot can drive 50 conversions to MV^•+^ per second before limitation by the number of photoexcited electrons (photon absorption rate is *ca.* 450 μM s^–1^). This corresponds to an average period of 20 ms for an MV^2+^ to attach to the carbon dot, accept an electron to form MV^•+^, then escape the solvent cage.

MV^2+^ adsorption competes with other adsorbing species, such as the SED and its oxidized counterparts, together with MV^•+^ that has not been able to escape the solvent cage, given the negatively charged surface of the carbon dots.

### Effect of EDTA Concentration

**Figure 4:**
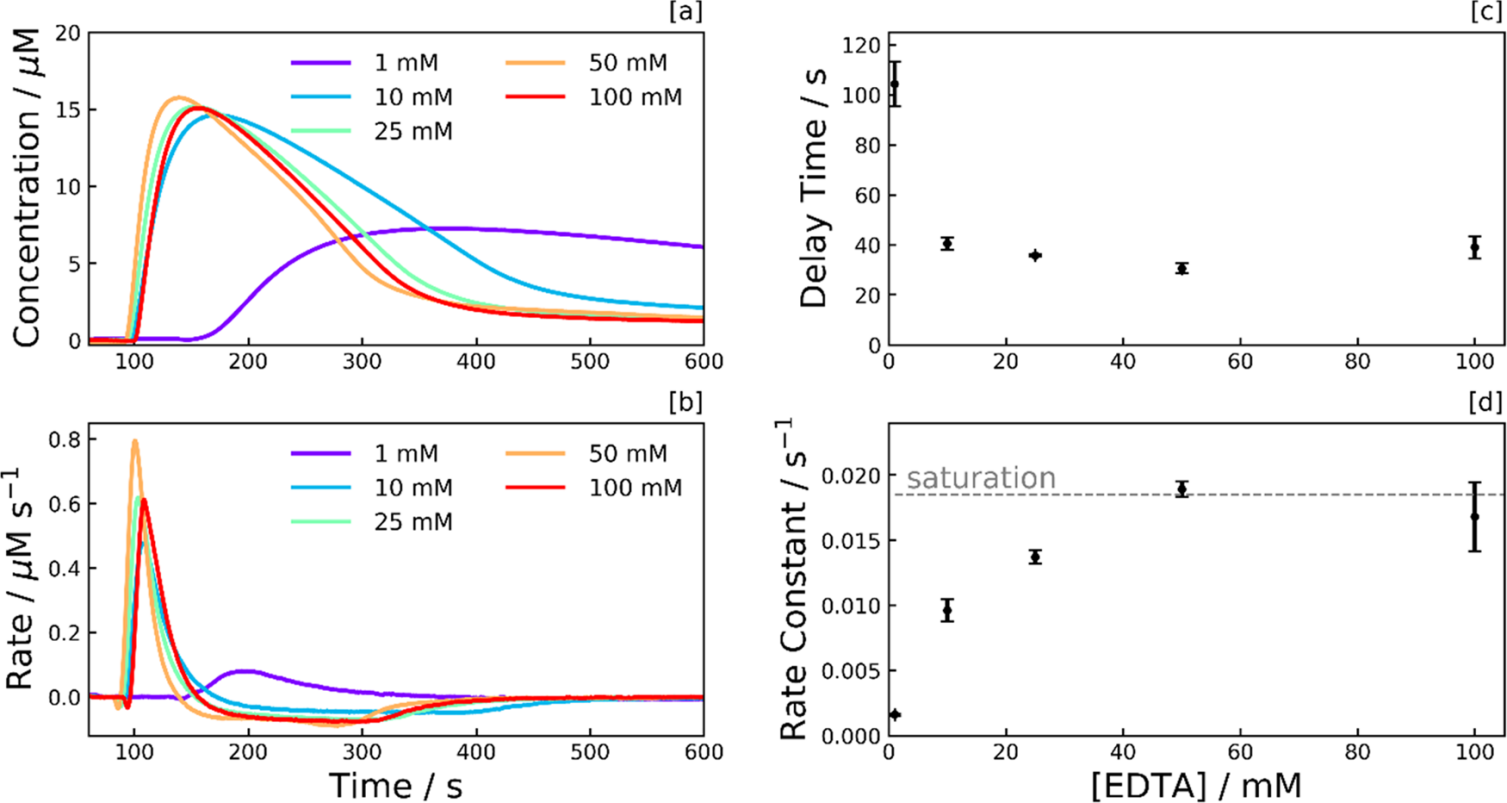
Photoreduction dependence on EDTA concentration. [a] MV^•+^ concentration, and [b] rate profiles, illustrating the dependence of the reaction kinetics on EDTA concentration. The MV^•+^ concentration was calculated from the UV–vis absorption peak at 600 nm. Each sample consists of EDTA with 0.5 g L^–1^ aCDs and 40 μM MV^2+^ in an aqueous pH 6 phosphate buffer. Samples were subject to continuous UV irradiation (λ = 365 nm) from *t* = 60 s at an irradiance of 90.2 mW cm^–2^. [c] Delay time and [d] photoreduction rate constant, both plotted as a function of EDTA concentration, with error bars signifying the standard deviation of at least triplicates.

To confirm whether the SED (EDTA) was rate-limiting and to validate our pseudo-first-order rate equation eq 9, the concentration of EDTA was varied between 1 and 100 mM. This set of samples was prepared with 0.5 g L^–1^ aCD and 40 μM MV^2+^ in an aqueous pH 6 phosphate buffer, loaded into the HC-PCF, and subsequently subjected to UV excitation as before. The corresponding concentration and rate profiles are shown in Figure 4a,b.

The delay time is relatively consistent in the 10–100 mM range (Figure 4c), with a significant increase noticeable at 1 mM. This behavior suggests the delay time is linked to the electron donation phenomenon to the CDs, and EDTA limits this phenomenon at 1 mM. Additional experiments substituting the CDs for the photosensitizer [Ru(bpy)_3_]^2+^ found that the delay time was not intrinsic to the CDs. We hypothesize that although great efforts were taken to purge oxygen in our measurement samples, a small amount of residual oxygen trapped at the photosensitizer surface must be depleted before the MV^2+^ photoreduction can begin. This residual oxygen is depleted by accepting photoexcited electrons from the CD, with a SED required to fill the remaining holes in the CD valence band.

The concentration profiles of the 25, 50, and 100 mM measurement runs are similar in Figure 4a, with the photoreduction rate constant starting to saturate above 25 mM in Figure 4d. This suggests there is a sufficient reservoir of EDTA above 25 mM for the photoreduction not to be rate-limited by the SED, and as such, we can attribute the rate-limiting step to photon absorption when the [EDTA] >25 mM. This exceeds the aCD concentration by 10^4^ times. However, given EDTA competes with MV^2+^ to access the CD surface, excessively large EDTA concentrations may counter-productively reduce the photoreduction rate constant. It is challenging to deduce whether this is the case at 100 mM within the margins of error.

The 1 and 10 mM samples appear to be limited by the EDTA reservoir, and the depletion rate is noticeably lower at these concentrations (Figure 4a,b). The decrease in the rate of depletion at 1 and 10 mM is a result of less CD^–^ being available to reduce MV^•+^ to MV^0^. Given the EDTA ions are negatively charged, the immediate surroundings of the negatively charged CD surface likely consist of an inner sphere of positively charged methyl viologen species and an outer sphere of negatively charged EDTA. Therefore, the electron transfer from the CD to the MV^2+^ is likely an inner-sphere electron transfer, and the electron donation from the EDTA to the CD is likely an outer-sphere transfer event.

### Effect of Irradiance

**Figure 5:**
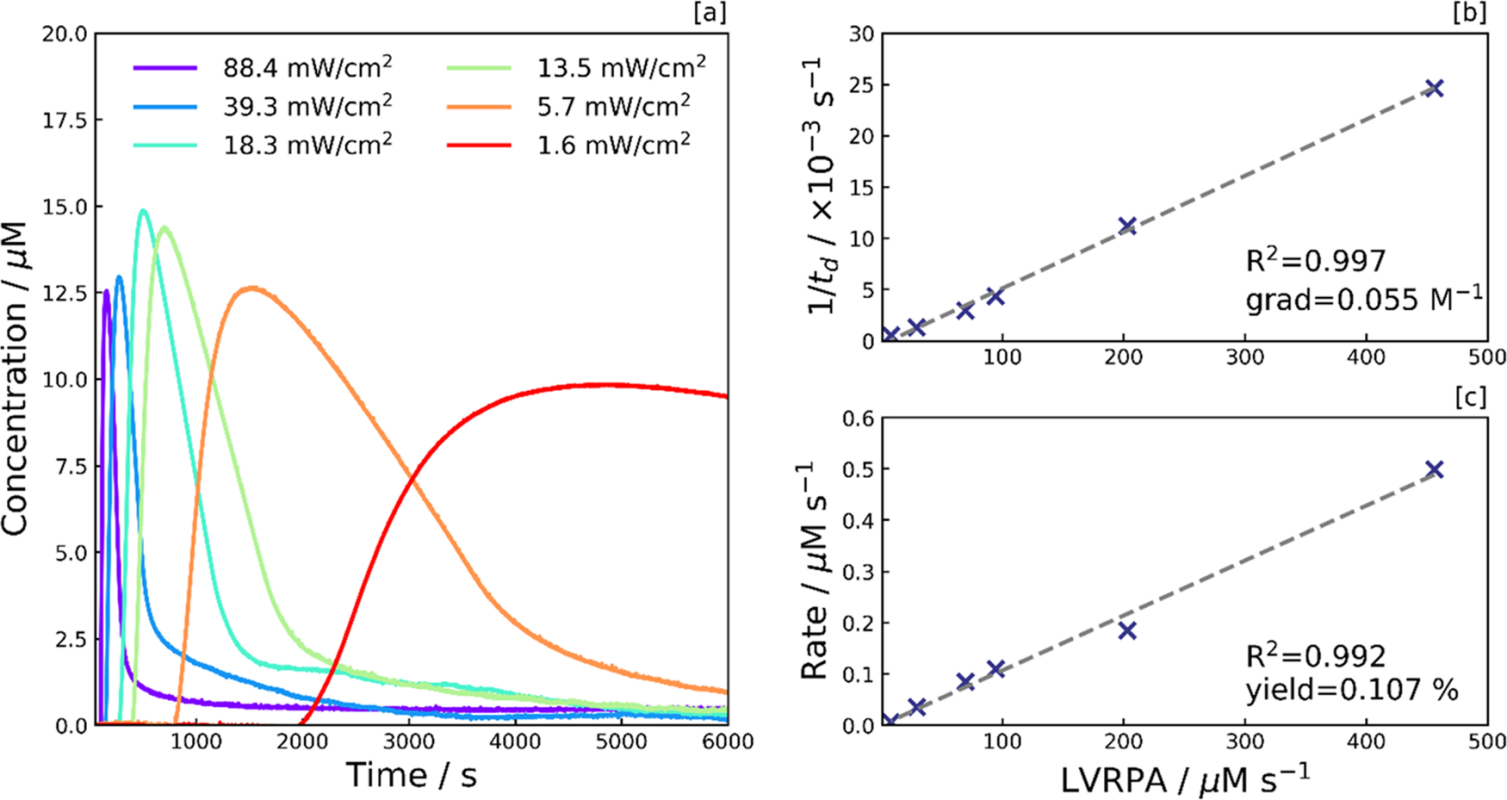
Photoreduction dependence on the local volumetric rate of photon absorption (LVRPA). [a] MV^•+^ concentration profile illustrating the dependence of the reaction kinetics on irradiance. The MV^•+^ concentration was calculated from the UV–vis absorption peak at 600 nm. Each sample consists of MV^2+^ with 0.5 g L^–1^ aCDs, 40 μM MV^2+^, and 0.1 M EDTA in an aqueous pH 6 phosphate buffer. Samples were subject to continuous UV irradiation (λ = 365 nm) from *t* = 60 s. [b] Delay time (inverse) and [c] photoreduction rate, both plotted as a function of LVRPA.

The irradiance dependence on the photoreduction reaction kinetics was explored using 6 different irradiances in the range 1.6–88.4 mW cm^–2^ with aCDs, MV, and EDTA. Reaction kinetic profiles were used to determine whether the photoreduction process was photon-limited and to calculate a quantum yield. This set of samples was made up of 0.5 g/L aCD, 40 μM MV^2+^, and 0.1 M EDTA in an aqueous pH 6 phosphate buffer. The results are shown in Figure 5a.

Irradiances, *I*, were measured using a Thorlabs S130 photodiode at the same distance below the optical diffuser as the HC-PCF (1 cm), with the values converted to an LVRPA by considering the CD absorption coefficient, ε_λ_, (see Supporting Information, Figure S4a) together with the CD concentration, [CD], and the excitation wavelength, λ, in eq 11.
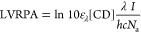
11

It is worth noting that adjustments to the irradiance are equivalent to adjustments to the CD concentration, as both quantities are directly proportional to the LVRPA. As such, the effect of increasing the irradiance on the shape of the concentration profile is similar to increasing the CD concentration, as depicted in Figure 2a. However, high LVRPA regimes are challenging to reach with highly concentrated solutions of CDs. At high [CD], viologen photoreduction cannot be analyzed in the fiber due to substantial absorption at 600 nm from the CDs over the 20 cm optical path length. In these cases, high LVRPA regimes can be accessed by increasing the irradiance.

The delay time (*t_d_*) was found to be inversely proportional to the LVRPA (Figure 5b), suggesting the delay time phenomenon is also light-driven (in addition to being dependent on CD concentration). The strong correlation supports the hypothesis that MV^2+^ photoreduction begins once a fixed amount of residual oxygen in the solution is depleted. A clear linear trend is observed between LVRPA and the initial photoreduction rate, indicating that the photoreduction process is indeed photo-limited, giving a quantum yield of 0.107% (see Figure 5c). This low yield relates to the fast relaxation of photoexcited CDs on the picosecond timescale, with only a small proportion of photoexcited CDs remaining long enough to take part in an electron transfer to the viologen dication.^15^

### Comparing Carbon Dots, Sacrificial Electron Donors, and Different Viologens

**Figure 6:**
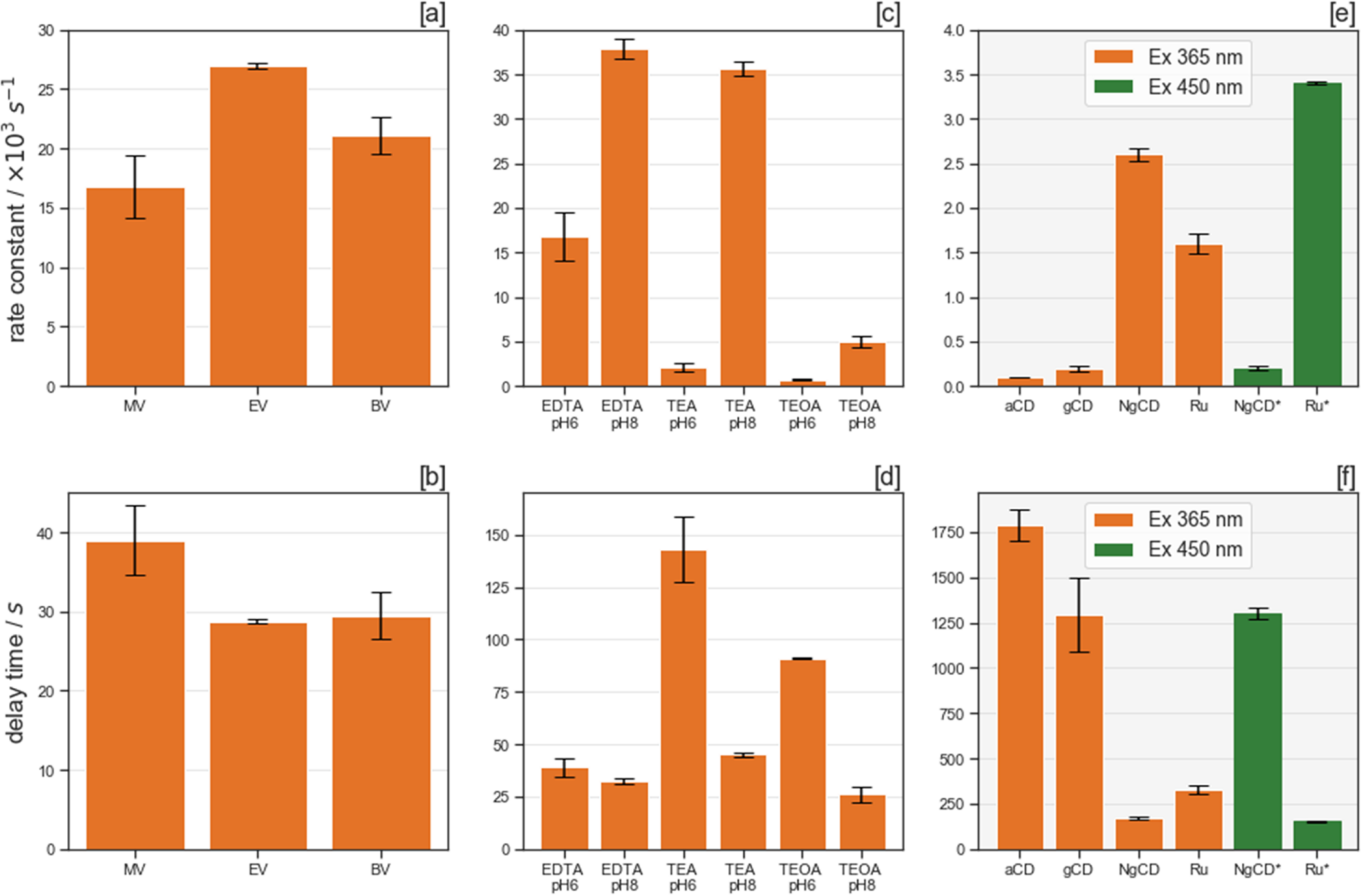
Photoreduction dependence on individual photosystem components. [a, b] Varying the viologen species: [a] photoreduction rate constants and [b] delay time. Each sample consists of 40 μM XV^2+^, 0.1 M EDTA, and 0.5 g L^–1^ aCD. [c, d] Varying the sacrificial electron donor and pH: [c] photoreduction rate constants and [d] delay time. Each sample consists of 40 μM MV^2+^, 0.1 M SED, and 0.5 g L^–1^ aCD. [e, f] Varying the photosensitizer type and excitation wavelength: [e] photoreduction rate constants and [f] delay time. Each sample consists of 40 μM MV^2+^, 0.1 M EDTA, and 0.01 g L^–1^ light absorber. Samples were subject to continuous UV irradiation (λ = 365 nm, 88.4 mW cm^–2^) or blue irradiation (λ = 450 nm, 99.1 mW cm^–2^). Ru = [Ru(bpy)_3_]^2+^, with Ru* and NgCD* denoting excitation at 450 nm. Error bars signify the standard deviation of at least triplicates.

The concentration sweeps of each component in the photosystem provide insight into the reaction kinetics of CD-driven photoreduction and support the reaction scheme presented. However, to further corroborate and understand the reaction mechanism, individual components of the photosystem were altered to probe their effects on the photoreduction rate constant and delay time, with the results shown in Figure 6.

Small changes in the molecular structure of the viologen species were used to gain insight into the electron transfer process at the surface of aCDs, with EDTA as the sacrificial electron donor (Figure 6a,b). The viologens studied comprised MV, EV, and BV, each expected to form electrostatically bound complexes together with aCDs, but with modified binding affinities.

Based on the redox potentials of methyl viologen (*E*^MV^2+^/MV^•+^^ = −0.446 V *vs* NHE),^47^ ethyl viologen (*E*^EV^2+^/EV^•+^^ = −0.449 V *vs* NHE),^47^ and benzyl viologen (*E*^BV^2+^/BV^•+^^ = −0.359 V *vs* NHE),^47^ the photoreduction rate should be fastest for BV^2+^, followed by MV^2+^ then EV^2+^ considering the average excited-state potential of COOH-functionalized CDs is −0.55 V *vs* reversible hydrogen electrode at pH 5.^48^ However, we do not observe this trend in Figure 6a, with electron transfer being the fastest for EV^2+^. We hypothesize that competing factors, such as the cage escape process and CD binding affinity, contribute to the reaction rate. For example, the bulkiness of the BV^2+^ species restricts the ease of cage escape.

The effect of varying the SED was investigated with EDTA, TEA, and TEOA at pH 6 and pH 8, in conjunction with aCDs and MV. Changes in the molecular structure of the SEDs were used to understand the depletion of XV^•+^ observed in all our measurements. Adjustments to pH also allowed us to tune the electron-donating ability of the SED.

The electron donor has a significant effect on both the photoreduction rate constant (Figure 6c) and delay time (Figure 6d). EDTA is well known as an electron donor that can operate in neutral conditions, with TEA and TEOA working best under alkaline conditions. The electron donation to CD* in reaction 2 is pH sensitive, with more basic conditions lessening the ability of the SED to donate electrons to CD*. Therefore, increasing the pH results in a greater rate of MV^•+^ production with all of the electron donors tested. The poor performance of TEA and TEOA at pH 6 is attributed to the inferior electron-donating ability of the protonated forms of TEA and TEOA (TEA p*K*_a_ = 10.7, TEOA p*K*_a_ = 7.9).^14^ EDTA displays an improved performance as its relevant p*K*_a_ value is 6.1,^14^ with both singly protonated and doubly protonated forms of EDTA coexisting at pH 6 in solution. It is surprising to see a ×7 higher photoreduction rate with TEA over TEOA, given that TEOA has a lower p*K*_a_. We postulate that the cage escape efficiency is faster with TEA over TEOA, thus producing the radical monocation more rapidly.

Overall, the delay time is dependent on the type of SED used (Figure 6d, Supporting Information Figure S10). At pH 6, TEA and TEOA have considerably longer delay times before the onset of viologen photoreduction. This is linked to the poor electron-donating ability of both these SEDs at pH 6, resulting in a longer amount of time to remove residual oxygen in the sample. When viologen photoreduction was performed at pH 8 with the different SEDs, the delay times are observed to be more similar, meaning that each SED was not inactivated under slightly alkaline conditions.

The performance of the photosensitizer was probed by comparing the rate of photoreduction of aCDs, gCDs, and NgCDs. NgCDs were benchmarked against [Ru(bpy)_3_]^2+^ under UV 365 nm and blue 450 nm irradiation. For this set of measurements, methyl viologen was utilized as the electron acceptor and EDTA as the electron donor (Figure 6e,f).

By comparing CDs at the same mass loading (10 mg L^–1^), we find that NgCDs perform the best out of the CDs by a considerable margin—with a ×10 higher photoreduction rate observed over its un-doped graphitic counterpart (Figure 6e). This increase corroborates well with the increase in reported turnover frequencies (TOF) for proton reduction when gCDs and NgCDs are used in conjunction with the molecular [Ni(P_2_N_2_)_2_]^2+^ DuBois-type catalyst (NiP).^5^ Given that the size distributions of both gCDs and NgCDs are similar,^5^ the difference in delay time cannot be attributed to a difference in the number density of CDs. The absorption coefficients of both gCDs and NgCDs are also similar (see Supporting Information, Figure S4a), which suggests that the excited-state lifetime differentiates the two. Therefore, we conclude that nitrogen doping promotes a greater proportion of long-lived species, which is also corroborated by transient absorption spectroscopy measurements.^15^

### Benchmarking

Benchmarking against [Ru(bpy)_3_]^2+^ at the same mass loading shows that although NgCDs drive higher rates of reaction under UV (365 nm) irradiation, [Ru(bpy)_3_]^2+^ performs best under blue (450 nm) irradiation (Figure 6e). This is partly because NgCDs have a ×2.3 lower absorbance at 450 nm, whereas [Ru(bpy)_3_]^2+^ has ×3.1 higher absorbance at this wavelength (see Supporting Information, Figure S4a). Consequently, a ×7.1 increase in rate is expected by changes in UV–vis absorption. Applying a small correction to account for the different photon fluxes incident with the 365 nm source and 450 nm sources, we arrive at an expected increase of ×9.8. However, we observe a ×13 increase in rate, highlighting the superior ability of [Ru(bpy)_3_]^2+^ in donating an electron to MV^2+^ in this photosystem. This is thought to be due to the extended excited-state lifetime of [Ru(bpy)_3_]^2+^ over the NgCDs, as the 450 nm transition corresponds to the metal-to-ligand charge transfer (MLCT) excited state that is ideally suited for facilitating outer-sphere electron transfer to substrates or co-catalysts.

### Effect of CD Surface

To interrogate the influence of CD surface charge on the rate constant and delay time, the performance of aCDs with protonated (COOH moieties), deprotonated (COO^–^ moieties) and tertiary-capped amine (NMe_2_^+^) surfaces were compared under the same conditions (0.5 g L^–1^ aCDs, 40 μM MV, 0.1M EDTA, pH 6).

**Figure 7:**
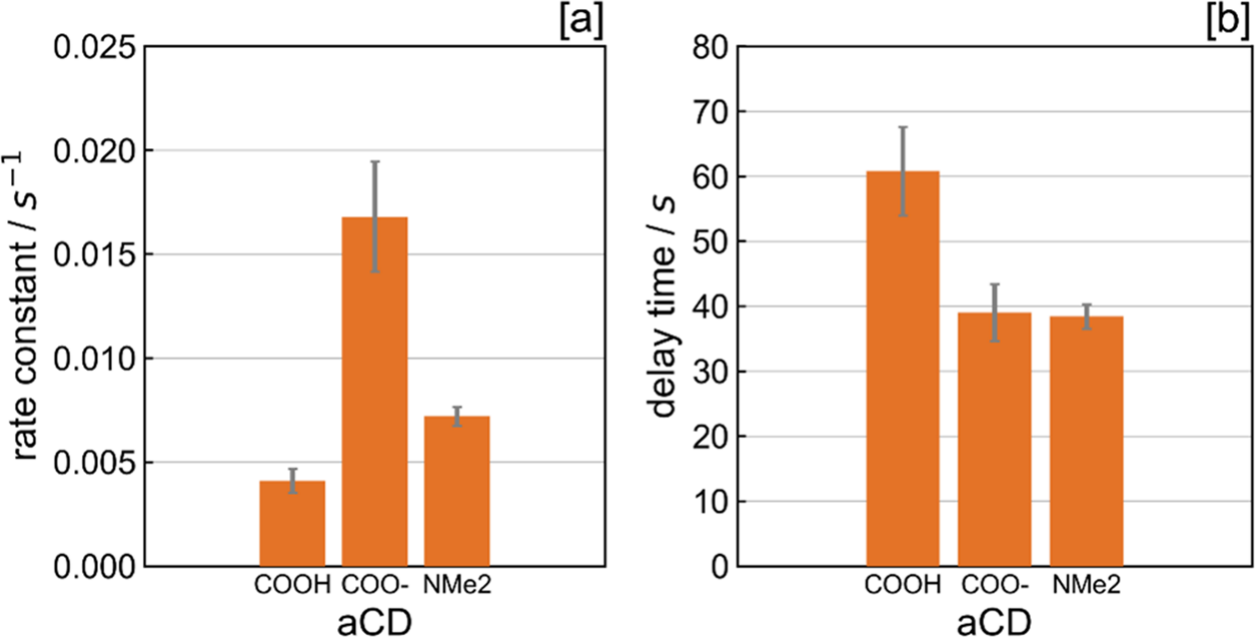
Comparison of protonated, deprotonated, and tertiary amine-capped aCDs. [a] photoreduction rate constant and [b] delay time. Each sample consists of 40 μM MV^2+^, 0.1 M EDTA, and 0.5 g L^–1^ aCD. Samples were subject to continuous UV irradiation (λ = 365 nm, 88.4 mW cm^–2^). Error bars signify the standard deviation of at least triplicates.

Our findings (Figure 7, Supporting Information Figure S12) show that a negative CD surface charge, and subsequent electrostatic attraction to MV^2+^, is advantageous for improving the rate of electron transfer, even overcoming the requirement for frontier orbitals to be largely present on the aCDs to facilitate the electron transfer to MV^2+^. This is corroborated by X-ray Absorption Spectroscopy (XAS) data.

X-ray absorption (XA) spectra of all three types of aCDs at the carbon K-edge (see Supporting Information, Figure S9), show a much lower contribution from transitions from C1s to π*_C=C_ (285.0 eV) and π*_C=O_ (288.6 eV) bonds in both the deprotonated and tertiary-capped amine aCDs, relative to their protonated analogue. The higher peak observed at the O K-edge XA spectra for the transition from O1s to π*_C=O_ (532.2 eV—see Supporting Information, Figure S9) for protonated aCDs also validates this further. Overall, this implies that the electrostatic attraction between deprotonated aCDs and MV^2+^ facilitates increased rates of electron transfer despite the much lower proportion of frontier π/π*orbitals in comparison to the protonated aCDs. In previous work, it was shown that electrons excited to π*_C=C_ in aCDs cannot be transferred to water due to the amorphous nature of the core, in contrast to the graphitic CDs.^49^ Electronic states related to surface groups are therefore more likely to be involved in electron transfer. Interestingly, despite the lack of frontier π/π*orbitals on the tertiary-capped amine dots, they display a higher electron transfer rate constant than the protonated aCDs. This is most likely due to increased ion-dipole interactions between the solvent cage and slightly positive surface charge on the tertiary-capped amine dots, as evidenced by the C–NHMe_2_^+^ signal in their N K-edge XA spectrum (399.9 eV—Supporting Information, Figure S9). Such interactions would constrain the aCD-NMe_2_^+^ dots within the solvent cage, thus facilitating faster electron transfer rates (despite the likely electrostatic repulsion with MV^2+^) in comparison to the aCD-COOH, which will have a distinct lack of ion-dipole interactions.

## Conclusions

We have shown that comprehensive *in situ* spectrokinetic analysis can be performed on a CD-driven photoreduction reaction with a total cumulative sample volume of <1.5 mL. This study builds upon our previous proof-of-principle work demonstrating the use of HC-PCF microreactors for studying photochemistry.^31^ Systematic, low-volume screening of 29 reaction conditions enabled us to understand the complex interplays between each component in our homogeneous photosystem, consisting of an electron acceptor, electron donor, and photosensitizer. We highlight the significant role of the electron donor, specifically the level of protonation, in controlling the kinetics of these viologen photoreduction reactions. Optofluidic microreactors with real-time optical detection provide unprecedented insight into the reaction dynamics of photocatalytic systems, and once measurements are performed in continuous flow, they are likely to become part of the automated catalyst screening systems of the future.

## Data Availability

The data that support the findings of this study are openly available at the University of Cambridge Apollo Repository.^50^

## Supporting Information Available

Data analysis methodology; optical setup diagrams; HC-PCF transmission characterization; cuvette-based measurements; concentration profiles with different SEDs and aCD surface moieties; and XAS data (PDF)

## Abbreviations

aCD: amorphous carbon dot
BV: benzyl viologen
CD: carbon dot
EA: electron acceptor
EDTA: ethylenediaminetetraacetic acid
EV: ethyl viologen
gCD: graphitic carbon dot
HC-PCF: hollow-core photonic crystal fiber
LVRPA: local volumetric rate of photon absorption
MLCT: metal-to-ligand charge transfer
MV: methyl viologen
NgCD: nitrogen-doped graphitic carbon dot
NHE: normalized hydrogen electrode
PEEK: polyether ether ketone
TEA: triethylamine
TEOA: triethanolamine
TEY: total electron yield
TFY: total fluorescence yield
UV: ultraviolet
XAS: X-ray absorption spectroscopy
