# Supplementary material for: Low-Volume Reaction Monitoring of Carbon Dot Light Absorbers in Optofluidic Microreactors
Source: ACS Catal. 2023 Jun 26;13(13):9090–101. doi: 10.1021/acscatal.3c02212 (PMC10334427; doi:10.1021/acscatal.3c02212)
Supplement: Supplementary file 1 — cs3c02212_si_001.pdf [file cs3c02212_si_001.pdf]

SUPPORTING INFORMATION

Low-volume reaction monitoring of carbon dot light absorbers in optofluidic microreactors

Takashi Lawson,[a,b] Alexander S. Gentleman,[a,b] Ava Lage,[b] Carla Casadevall,[b] Jie Xiao,[c] Tristan Petit,[c] Michael H. Frosz,[d] Erwin Reisner,[b,*] and Tijmen G. Euser[a,*]

[a.] *NanoPhotonics Centre, Cavendish Laboratory, Department of Physics, University of Cambridge, JJ Thomson Ave, Cambridge CB3 0HE, United Kingdom.*
[b.] *Yusuf Hamied Department of Chemistry, University of Cambridge, Lensfield Road, Cambridge CB2 1EW, United Kingdom.*
[c.] *Helmholtz-Zentrum Berlin für Materialien und Energy GmbH, Albert-Einstein-Str. 15, 12489 Berlin, Germany*
[d.] *Max Planck Institute for the Science of Light, Staudtstr. 2, 91058 Erlangen, Germany.*

*Corresponding Authors: Tijmen G. Euser (te287@cam.ac.uk) and Erwin Reisner (reisner@ch.cam.ac.uk).

Contents

Supplementary Methods 2
Reaction Monitoring Data Analysis 2
Supplementary Figures 2
Figure S1 | 3D renderings of the pressure cells, excitation geometry and fiber-coupling optics. 2
Figure S2 | Transmission characteristics of the HC-PCF used in these experiments. 3
Figure S3 | Fiber transmission spectrum of the probe source used in these experiments. 3
Figure S4 | Absorption coefficients of carbon dots and $[Ru(bpy)_3]^{2+}$. 4
Figure S5 | Attenuated total reflectance infrared (ATR-IR) and Raman characterisation of all carbon dots studied. 4
Figure S6 | Cuvette-based UV-Vis absorption with the separate injection of each component. 5
Figure S7 | Cuvette-based UV-Vis with chemically reduced $MV^{\bullet+}$ titrated with aCDs. 6
Figure S8 | NMR data of chemically reduced $MV^{\bullet+}$ titrated with neutralised (COO^- terminated) aCDs. 7
Figure S9 | X-ray Absorption Spectroscopy (XAS) of aCDs with different surface moieties. 8
Figure S10 | Concentration profiles of $MV^{\bullet+}$ with EDTA, TEA and TEOA at [a] pH 6 and [b] pH 8. 9
Figure S11 | Scaling of the absorption coefficient of methyl viologen. 9
Figure S12 | Concentration profiles of $MV^{\bullet+}$ with different aCD surface moieties. 10
Supplementary Tables 10
Table S1 | Measurements of pH to accompany CD titration data presented in Figure S7. 10
References 11

Supplementary Methods

Reaction Monitoring Data Analysis

Each sample was loaded into the fiber microreactor and subject to UV (365 nm) or blue (450 nm) irradiation, following a 1-minute pause in the dark to ensure that the reference spectrum was stable and that the probe source (supercontinuum laser) did not drive the reaction. A short period after the excitation source is turned on, a rapid photoinduced absorption peaking at 600 nm is observed, indicating the formation of $XV^{\bullet+}$.[1]

Absorbance was converted to concentration by applying the Beer-Lambert Law with the reported values of absorption coefficients for MV ($1.3\text{x}10^4$ M^{-1} cm^{-1}), EV ($1.22\text{x}10^4$ M^{-1} cm^{-1}) and BV ($1.4\text{x}10^4$ M^{-1} cm^{-1}).[2,3] The average absorbance within a 10 nm wide band was taken for this conversion around the absorption peak at 600 nm unless stated otherwise (**Figure 1d**). The values of t_d and k were obtained by fitting a linear function to a short time interval following the formation of the $XV^{\bullet+}$ radical monocation, as shown in **Figure 1e**. This fitting method ensures that consumable reactants are in excess. Therefore, the gradient of the linear fitting function relates to the pseudo first order photoreduction rate constant, *k*, by **Equation S1**.

$$k = \frac{\frac{\mathrm{d}}{\mathrm{d}t}[\mathrm{XV}^{\bullet+}]_{t=t_0}}{[\mathrm{XV}^{2+}]_{t=t_0}} \qquad \text{(S1)}$$

The x-intercept of the fitted function was used to define the time at which electron transfers start, t_0, and thus define a delay period, t_d, following the excitation source being switched on.

Where rate profiles of the viologen radical monocation are presented, a Savitzky-Golay filter was applied to minimise undue noise whilst retaining peak heights.[4] The relevant parameters applied for filtering were a window size of 31 and polynomial order of 3. Savitzky-Golay filtering was not applied to the concentration and % conversion profiles presented.

Supplementary Figures

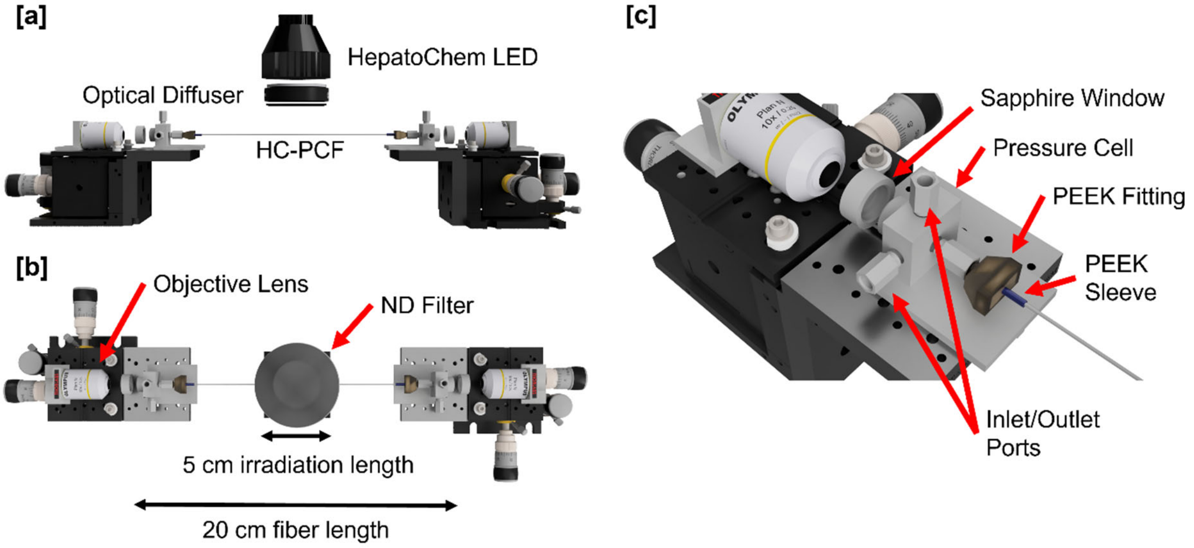

Figure S1 | 3D renderings of the pressure cells, excitation geometry and fiber-coupling optics. The 3D-printed neutral density (ND) filter stage and inlet/outlet tubing are omitted for clarity. [a] Side view showing the excitation source with optical diffuser above the HC-PCF. [b] Top view showing the square ND filters used to modify the irradiance, together with the 5 cm irradiation and 20 cm fiber lengths. [c] Magnified rendering of the pressure cell, showing how the pressure cell interfaces with the optics and fluidics. HC-PCFs were sealed using PEEK sleeves and commercially available microfluidic PEEK fittings.

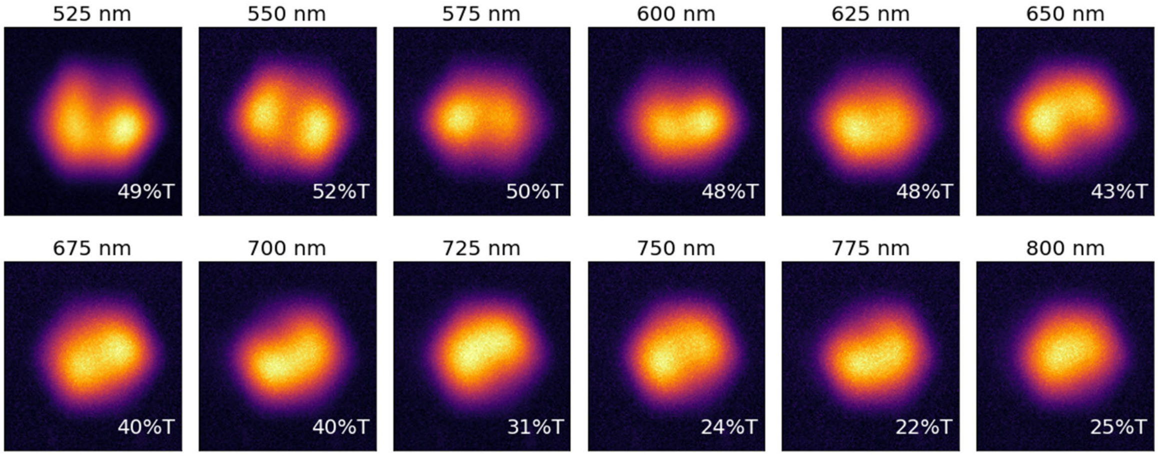

Figure S2 | Transmission characteristics of the HC-PCF used in these experiments. An NKT SuperK Extreme EXR-15 supercontinuum laser was efficiently coupled into a 20 cm length of kagomé-style HC-PCF infiltrated with water. The transmission was optimised at 600 nm. A wavelength sweep was performed from 525 nm to 800 nm (10 nm bandwidth) using an NKT SuperK Select tuneable filter. Linearly polarised optical modes are excited in the core of the fiber and are shown (LP_{01}, LP_{11}). Transmission % was calculated by comparing the optical power before the in-coupling objective and after the out-coupling objective using a Thorlabs S130 photodiode. The drop-off in transmission above 700 nm is attributed to the near-infrared absorption of water.

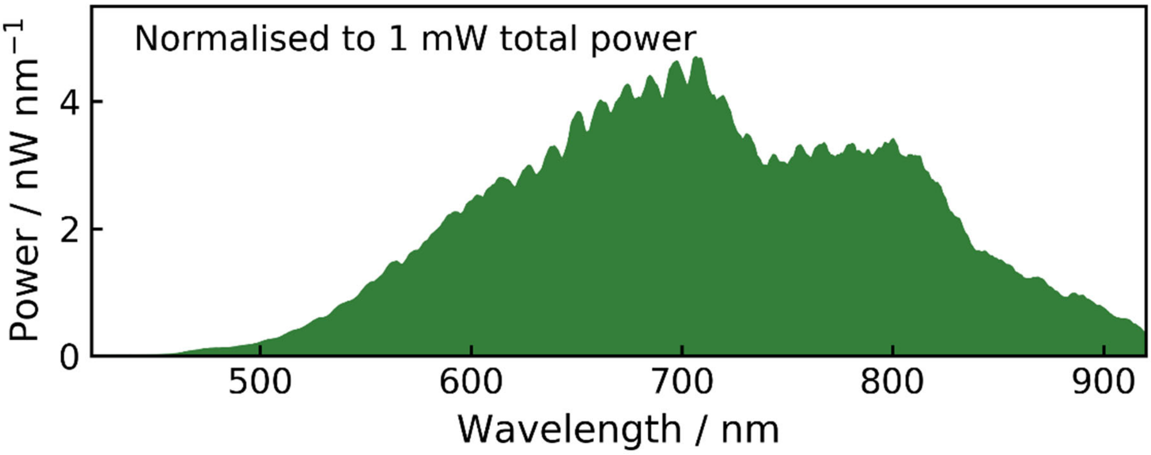

Figure S3 | Fiber transmission spectrum of the probe source used in these experiments. A 20 cm length of kagomé-style HC-PCF was infiltrated with water and the output from an NKT SuperK Compact supercontinuum laser was coupled into the HC-PCF. The normalised transmission spectrum is presented.

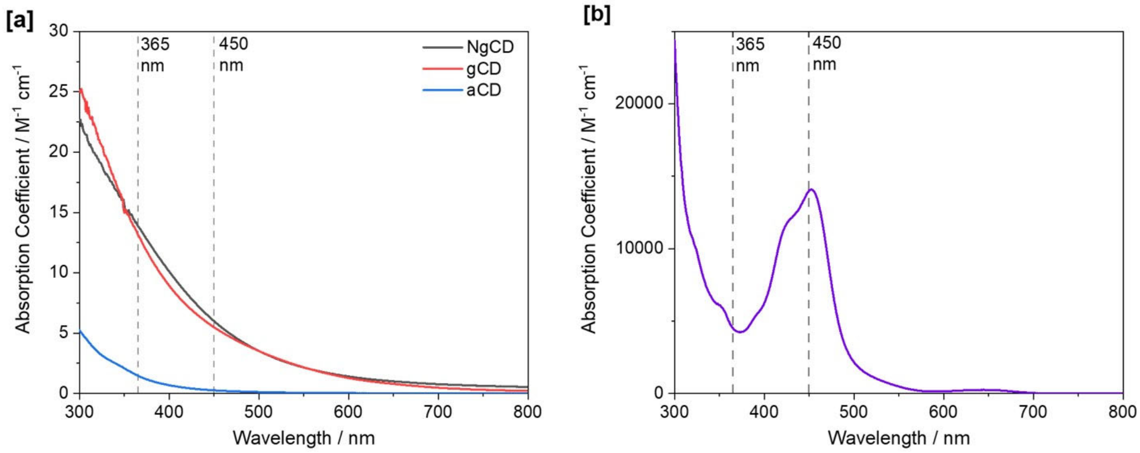

Figure S4 | Absorption coefficients of carbon dots and $[Ru(bpy)_3]^{2+}$. [a] Absorption profiles of amorphous, graphitic, and nitrogen-doped graphitic carbon dots (aCDs, gCDs and NgCDs). [b] Absorption profile of $[Ru(bpy)_3]^{2+}$. Absorption measurements were taken in an Agilent Cary 300 UV-Vis Spectrophotometer.

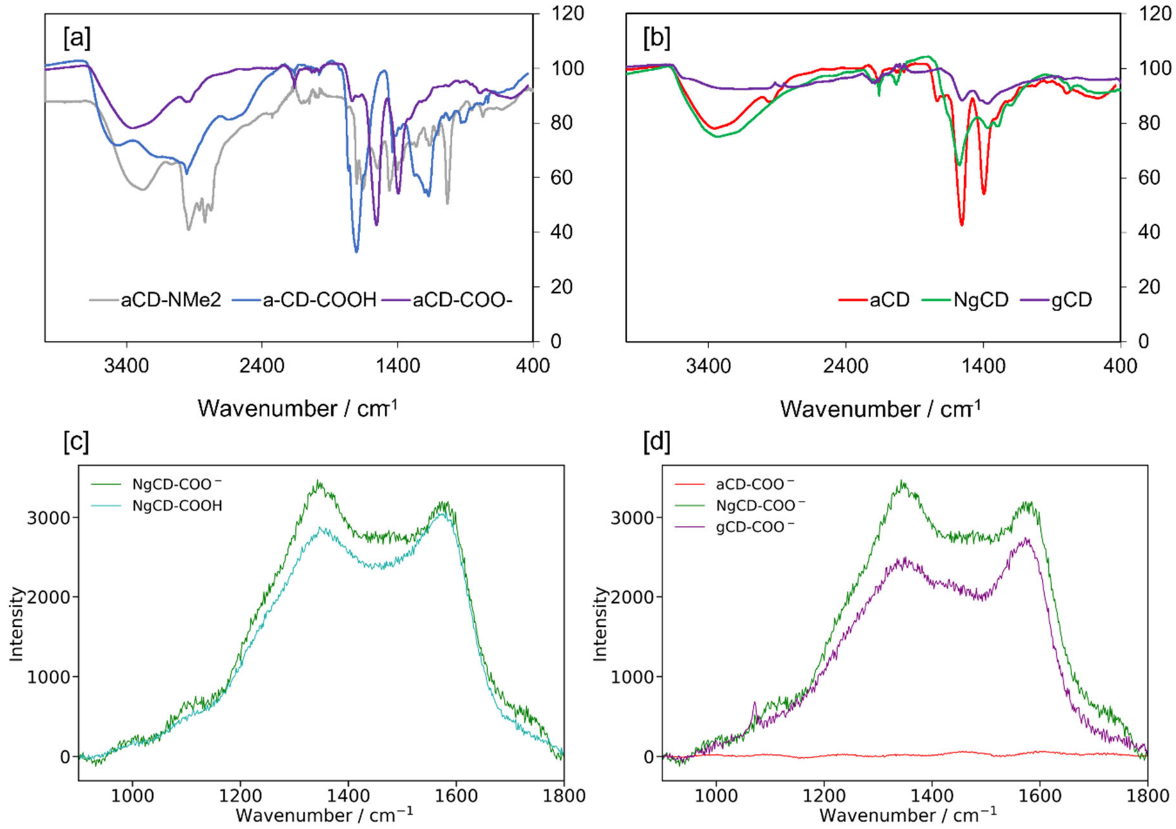

Figure S5 | Attenuated total reflectance infrared (ATR-IR) and Raman characterisation of all carbon dots studied. [a] ATR-IR spectra of amorphous carbon dots (aCDs) with different surface moieties. [b] ATR-IR spectra of neutralised (COO^- terminated) amorphous, graphitic, and nitrogen-doped graphitic carbon dots (aCDs, gCDs and NgCDs). [c] Raman spectra of protonated and neutralised (COO^- terminated) NgCDs, focussing on the D (1350 cm^{-1}) and G (1580 cm^{-1}) bands. [d] Raman spectra of neutralised aCDs, gCDs and NgCDs, focussing on the D and G bands.

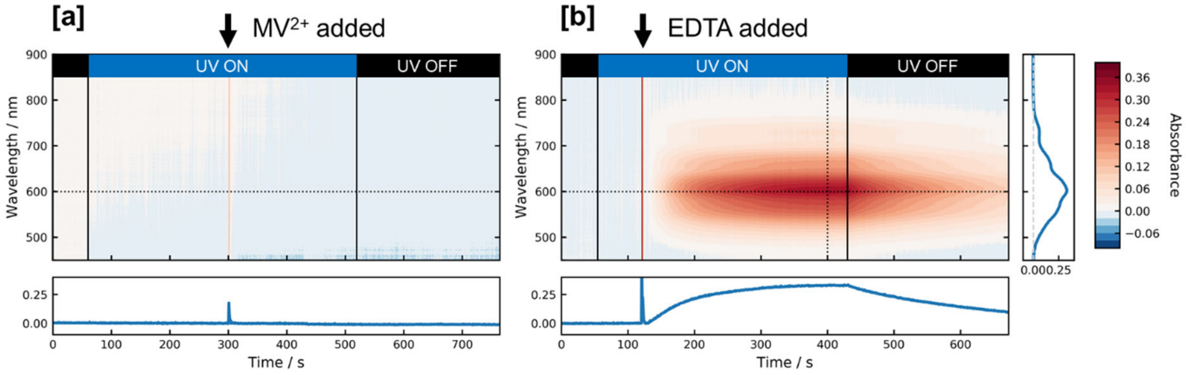

Figure S6 | Cuvette-based UV-Vis absorption with the separate injection of each component. A sample of 0.5 g/L amorphous carbon dots (aCDs) in an aqueous pH6 phosphate buffer was prepared in a gas-tight cuvette and purged under nitrogen for 15 minutes. A magnetic stirrer ensured sufficient mixing within the cuvette volume, with nitrogen overpressure maintained during the spectral measurements. Time-resolved absorption spectra of the sample are shown on [a] MV^{2+} addition and then [b] on the addition of EDTA. UV excitation and component addition times are as labelled. The aCD sample was subject to UV irradiation (λ = 365 nm, 174 mW/cm^2) at right angles to the collimated beam from the halogen source (Ocean Optics DH-2000) used to perform absorption spectroscopy. 0.1 mL of a 1.3 mM MV^{2+} stock solution was injected using a syringe at t = 300 s. The spectral measurement was then stopped at t = 750 s for the syringe to be cleaned and loaded with EDTA. The measurement was then restarted, and 0.1 mL of a 0.2 M EDTA stock solution was added at t = 120 s. A rapid absorption following EDTA addition was observed, due to the production of the MV radical monocation. The horizontal dotted lines in [a, b] correspond to the absorption time trace taken at 600 nm, and the vertical dotted line in [b] corresponds to the spectrum shown on the right at t = 400 s.

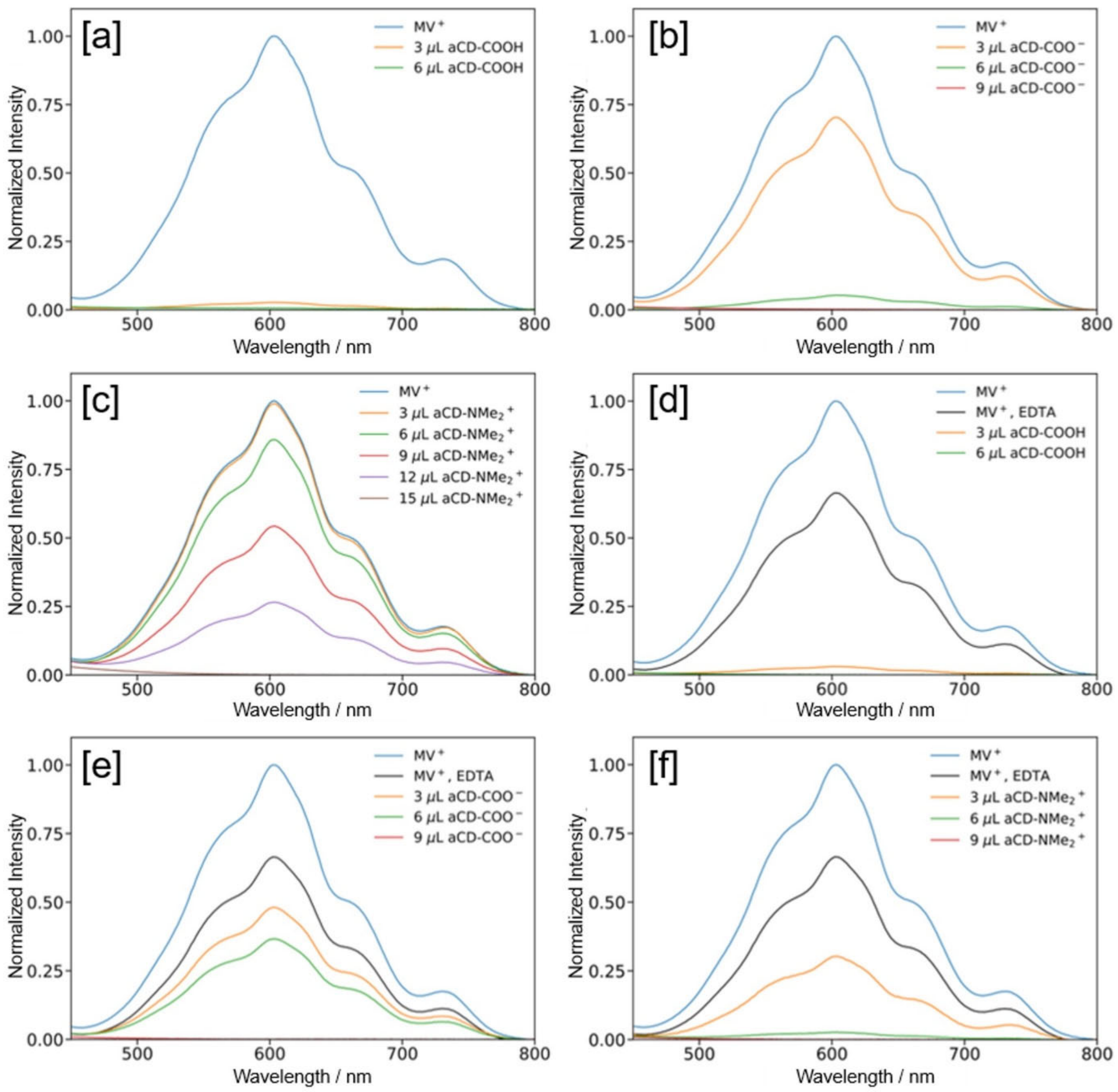

Figure S7 | Cuvette-based UV-Vis with chemically reduced $MV^{•+}$ titrated with aCDs. [a] COOH, [b] COO^{-} and [c] NMe_2 surface moieties. MV^{2+} (160 μM, 600 μl) was chemically reduced with sodium dithionite (16 mM stock, 9 μL, 1.5 equivalents) under nitrogen in a gas-tight cuvette. Then, reduced $MV^{•+}$ (160 μM, 600 μl) was titrated with various aCDs (16 μM, additions of 3 μl, 0.0005 equivalents/addition) under nitrogen at room temperature. The final concentration of aCD-COOH after 6 μL of additions was 0.16 μM, the final concentration of aCD-COO^{-} after 9 μL of additions was 0.24 μM and the final concentration of aCD-NMe_2 after 15 μL of additions was 0.39 μM. For the titrations with EDTA [d-f], EDTA (16 μM, 6 μL, 1 equivalent) was added to the reduced MV before titration with the aCDs. The spectra presented have the absorption contribution from the aCDs subtracted. Refer to Table S1 for pH measurements.

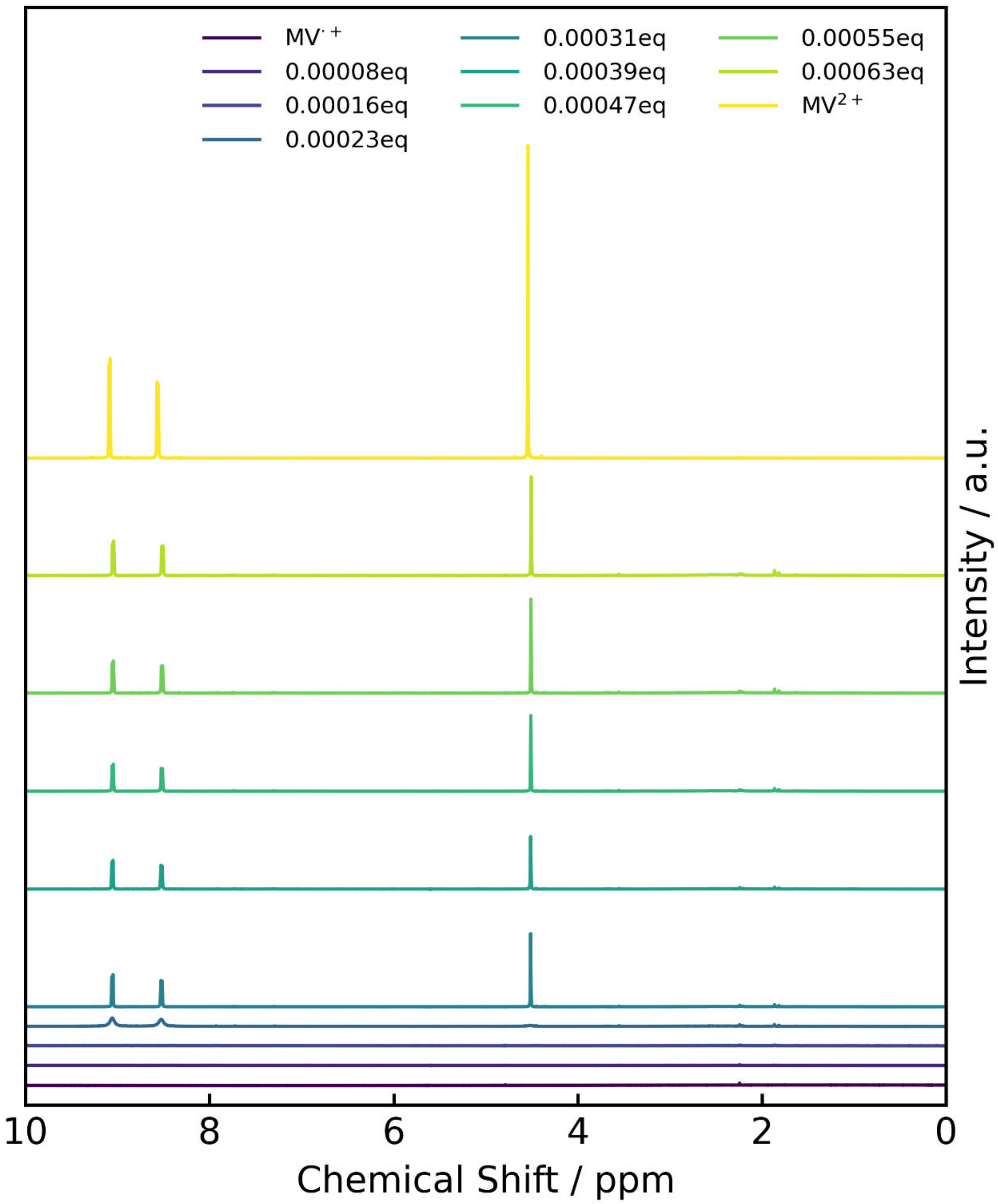

Figure S8 | NMR data of chemically reduced $MV^{\bullet+}$ titrated with neutralised (COO^- terminated) aCDs. MV^{2+} (16 mM, 600 ml) was chemically reduced with sodium dithionite (1.5 equivalents, 25 mM) in a wet glovebox under nitrogen in a young NMR tube. Then, reduced $MV^{+\cdot}$ (16 mM, 600 ml) was titrated with the aCDs under nitrogen at room temperature. Final concentration of aCDs was 10 µM (0.00063 eq).

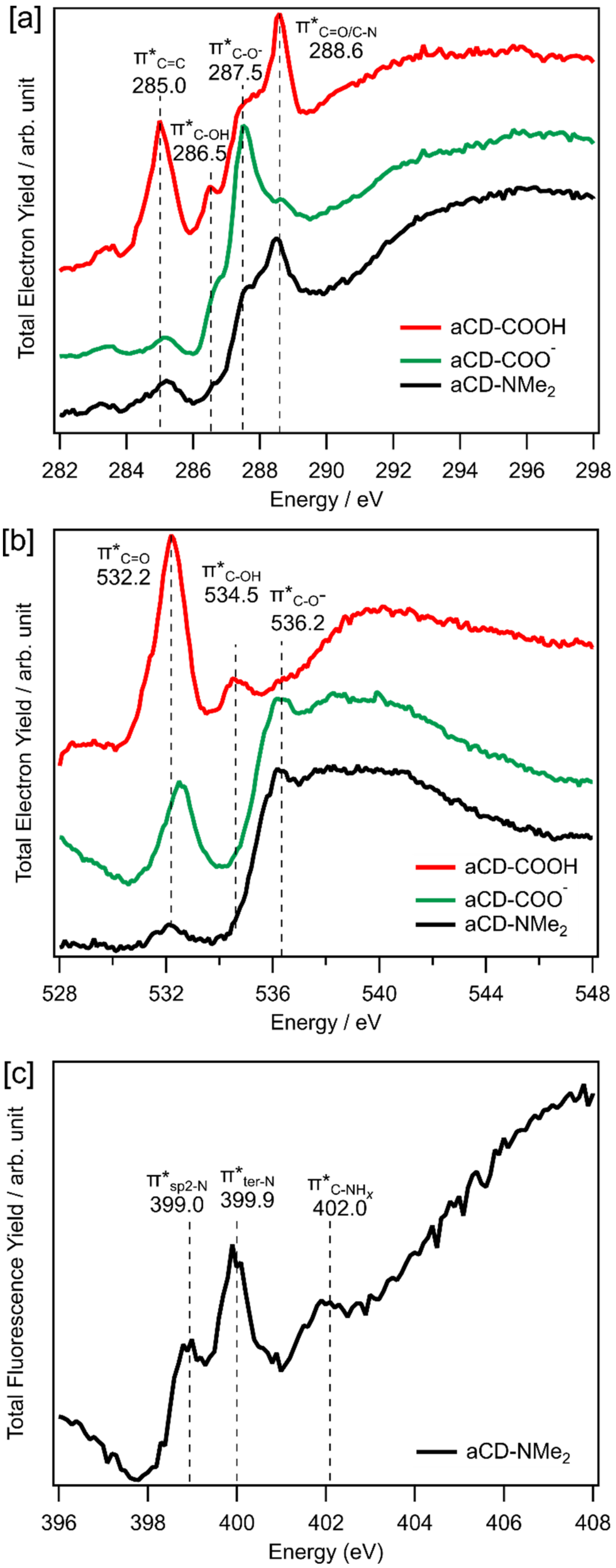

Figure S9 | X-ray Absorption Spectroscopy (XAS) of aCDs with different surface moieties. XAS at the [a] carbon, [b] oxygen and [c] nitrogen K-edges are shown for the different samples. The spectra are normalized to the pre- and post-edges and separated for clarity. The tentative attribution to the main features is labelled based on literature.[5,6] Sp^2-N refers to graphitic nitrogen and ter-N to tertiary amino groups.

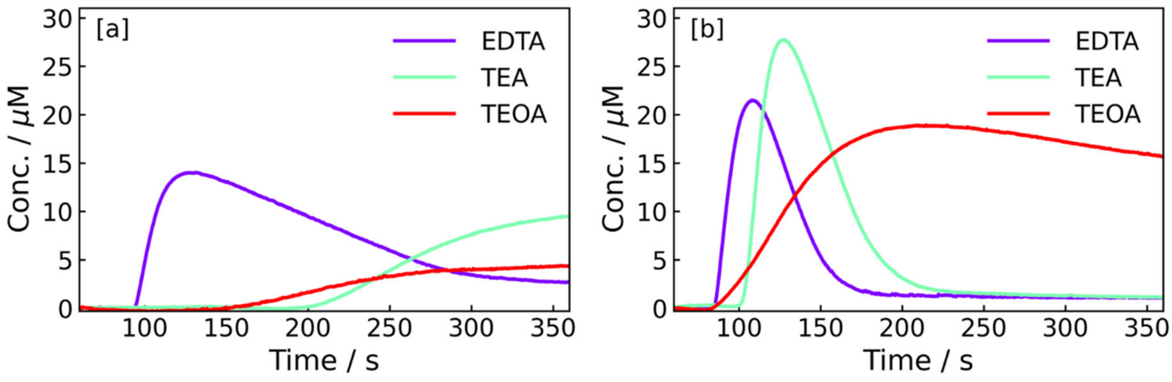

Figure S10 | Concentration profiles of $MV^{•+}$ with EDTA, TEA and TEOA at [a] pH 6 and [b] pH 8. Each sample consisted of 0.5 g/L aCDs with 40 μM MV^{2+} and 0.1 M of the sacrificial electron donor (SED) in an aqueous phosphate buffer (0.2M). Samples were subject to UV irradiation (λ = 365 nm, 88.4 mW/cm^2) from t = 60 s. The $MV^{•+}$ concentration was calculated from the UV-Vis absorption peak at 600 nm.

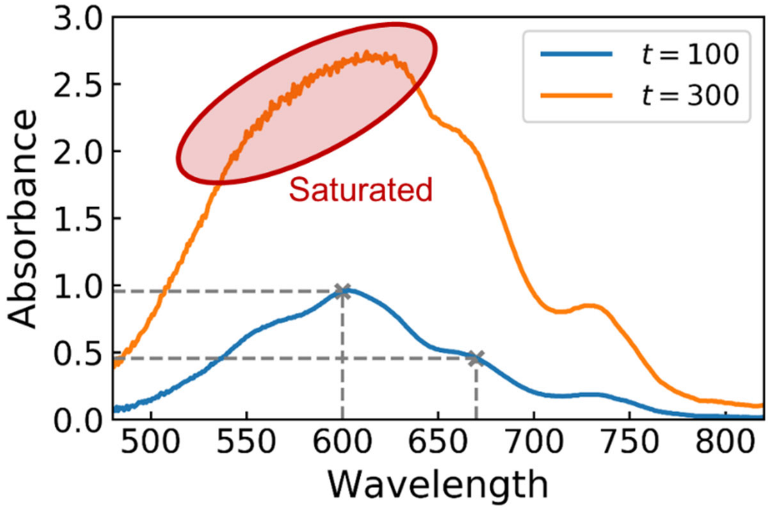

Figure S11 | Scaling of the absorption coefficient of methyl viologen. The absorbance at 600 nm and 670 nm was extracted at t = 100 s and the fraction was taken to scale the known absorption coefficient of methyl viologen at 600 nm. The absorption spectrum at t = 300 s shows that although the absorbance at 600 nm was saturated, the absorbance at 670 nm is not.

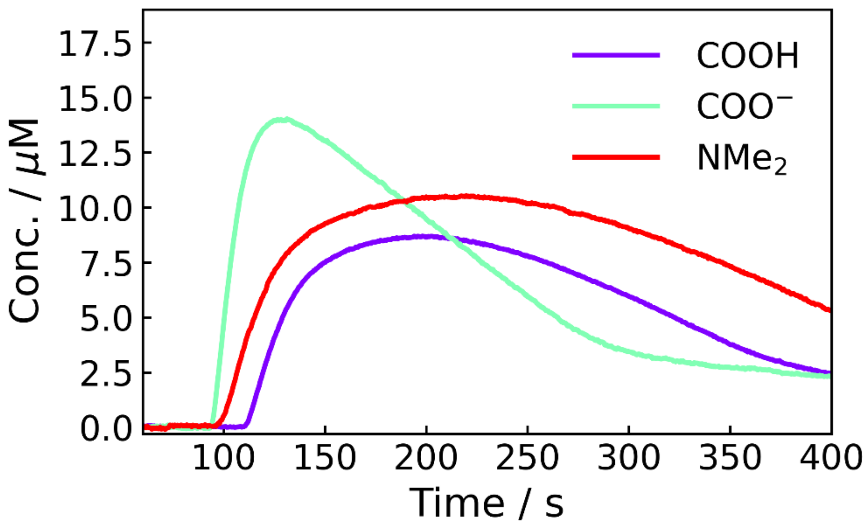

Figure S12 | Concentration profiles of $MV^{\bullet+}$ with different aCD surface moieties. Each sample consisted of 0.5 g/L aCDs with 40 μM MV^{2+} and 0.1 M EDTA in an aqueous phosphate buffer (pH 6, 0.2M). Samples were subject to UV irradiation (λ = 365 nm, 88.4 mW/cm^2) from t = 60 s. The $MV^{\bullet+}$ concentration was calculated from the UV-Vis absorption peak at 600 nm.

Supplementary Tables

Table S1 | Measurements of pH to accompany CD titration data presented in Figure S7. MV^{2+} (160 μM, 600 μl) was chemically reduced with sodium dithionite (16 mM stock, 9 μL, 1.5 equivalents) under nitrogen in a gas-tight cuvette. Then, reduced $MV^{\bullet+}$ (160 μM, 600 μl) was titrated with various aCDs (16 μM, additions of 3 μl, 0.0005 equivalents/addition) under nitrogen at room temperature. For the titrations with EDTA, EDTA (16 μM, 6 μL, 1 equivalent) was added to the reduced MV before titration with the aCDs.

**MV^{2+} with Sodium Dithionite<br>Titration starting at pH 6.67**			
**Volume (μL)**	**aCD-COOH**	**aCD-COO⁻**	**aCD-NMe_2^+**
3	4.29	6.84	7.19
6	3.93	6.96	7.51
9	-	7.01	7.70
12	-	-	7.80
15	-	-	7.89
**MV^{2+} with Sodium Dithionite and EDTA<br>Titration starting at pH 6.07**			
**Volume (μL)**	**aCD-COOH**	**aCD-COO⁻**	**aCD-NMe_2^+**
3	4.12	6.39	6.78
6	3.85	6.75	7.09
9	-	6.88	7.37

References

(1) Koehler, P.; Lawson, T.; Neises, J.; Willkomm, J.; Martindale, B. C. M.; Hutton, G. A. M.; Antón-García, D.; Lage, A.; Gentleman, A. S.; Frosz, M. H.; Russell, P. St. J.; Reisner, E.; Euser, T. G. Optofluidic Photonic Crystal Fiber Microreactors for In Situ Studies of Carbon Nanodot-Driven Photoreduction. *Anal Chem* **2021**, *93* (2), 895–901. https://doi.org/10.1021/acs.analchem.0c03546.

(2) Tsukahara, K.; Wilkins, R. G. Kinetics of Reduction of Eight Viologens by Dithionite Ion. *Journal of the American Chemical Society* **1985**, *107* (9), 2632–2635. https://doi.org/10.1021/ja00295a013.

(3) Imabayashi, S.-I.; Kitamura, N.; Tazuke, S.; Tokuda, K. The Role of Intramolecular Association in the Electrochemical Reduction of Viologen Dimers and Trimers. *Journal of Electroanalytical Chemistry and Interfacial Electrochemistry* **1988**, *243* (1), 143–160. https://doi.org/10.1016/0022-0728(88)85035-6.

(4) Savitzky, Abraham.; Golay, M. J. E. Smoothing and Differentiation of Data by Simplified Least Squares Procedures. *Analytical Chemistry* **1964**, *36* (8), 1627–1639. https://doi.org/10.1021/ac60214a047.

(5) Ren, J.; Weber, F.; Weigert, F.; Wang, Y.; Choudhury, S.; Xiao, J.; Lauermann, I.; Resch-Genger, U.; Bande, A.; Petit, T. Influence of Surface Chemistry on Optical, Chemical and Electronic Properties of Blue Luminescent Carbon Dots. *Nanoscale* **2019**, *11* (4), 2056–2064. https://doi.org/10.1039/C8NR08595A.

(6) Zhang, J.-R.; Ma, Y.; Wang, S.-Y.; Ding, J.; Gao, B.; Kan, E.; Hua, W. Accurate K-Edge X-Ray Photoelectron and Absorption Spectra of g-C 3 N 4 Nanosheets by First-Principles Simulations and Reinterpretations. *Physical Chemistry Chemical Physics* **2019**, *21* (41), 22819–22830. https://doi.org/10.1039/C9CP04573B.
